# *Citrus reticulata* Blanco: A Review on Chemical Composition and Biological Activities

**DOI:** 10.1002/cbdv.202503156

**Published:** 2026-01-08

**Authors:** José Walber Gonçalves Castro, Geane Gabriele de Oliveira Souza, José Galberto Martins da Costa, Fabíola Fernandes Galvão Rodrigues

**Affiliations:** ^1^ Post Graduate Program in Biological Chemistry Department of Biological Chemistry Regional University of Cariri (URCA) Crato Ceará Brazil

**Keywords:** biological activities, *Citrus reticulate*, essential oil, flavonoids

## Abstract

This review compiled data on the chemical composition and biological activities of *Citrus reticulata* (Rutaceae) from 49 articles published between 2014 and 2024 in the Science Direct, PubMed, and SciELO databases. The fruit peel stands out for its abundance of monoterpenes and sesquiterpenes, especially limonene (up to 85.7%), and methoxylated flavonoids such as nobiletin, tangeretin, and hesperidin, which are known for their antioxidant, anti‐inflammatory, and antimicrobial activities. The essential oil demonstrated larvicidal activity against *Aedes aegypti* and action against *Leishmania amazonensis*. It is concluded that the peel is rich in metabolites with high therapeutic and industrial potential, and further studies are recommended on mechanisms of action and applications in food, cosmetics, insecticides, and fertilizers.

## Graphical abstract

This review compiles chemical information and biological activities from 49 studies on Citrus reticulata. The fruit peels stand out for their high content of methoxylated monoterpenes and flavonoids with antioxidant, anti‐inflammatory, and antimicrobial activities. The essential oil exhibits larvicidal and leishmanicidal action, suggesting potential therapeutic and industrial applications.

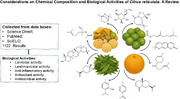

## 1 Introduction

Brazilian citriculture stands out as one of the largest producers worldwide. Orange trees, tangerine trees, acid limes, and true lemons are the main types of *citrus* cultivated in Brazil. The most recognized citrus varieties include sweet orange (*Citrus sinensis*), tangerine (*Citrus reticulata*), lemon (*Citrus limon*), grapefruit (*Citrus paradisi*), and pummelo (*Citrus grandis* or *Citrus maxima*) [1], which predominantly influence various systems such as the immune, reproductive, cardiovascular, and central nervous systems [2].

*C. reticulata* Blanco belongs to the Rutaceae family, which consists of more than 1600 shrub and small tree species and is native mainly to tropical, subtropical, and temperate countries. In Brazil, 33 genera and approximately 192 species are described [3]. Seasonal flowering is primarily controlled by environmental conditions, which directly influence its chemical composition; environmental cues regulate the initial development of the fruits and, therefore, the plants’ ability to reproduce multiple times throughout their lifespan, thus altering the biological activities of the species. However, in tropical areas, flowering becomes continuous, determined by the availability of moisture from sufficient rainfall or water supply, whereas in temperate regions or those with drought periods, flowering occurs at the onset of winter [4, 5, 6].

*C. reticulata* is an excellent source of vitamins C and A, proteins, dietary fibers, and essential minerals, including calcium, potassium, phosphorus, and magnesium, in addition to small amounts of B‐complex vitamins. On average, 100 g of tangerine is composed of 85.2 g of water, 13.34 g of carbohydrates, 0.81 g of protein, 0.38 g of dietary fiber, and 0.31 g of fat. The B‐vitamin complex contributes to infection prevention, promotes cellular health, and maintains adequate energy levels, nerve functions, hormone and cholesterol production, and also favors cardiovascular health [7, 8, 9].

The chemical components found in the peels and leaves of *C. reticulata* have been highlighted for their antinociceptive properties, as is the case with methyl‐*N*‐methyl anthranilate. Moreover, terpenoids, flavonoids, and phenolic compounds play an important role in defense against insects and microbial attacks. Noteworthy components include aldehydes, limonene, ketones, esters, alcohols, terpenes, β‐myrcene (**8**), 3‐carene (**16**), and α‐pinene (**5**), which provide, besides biological activities, the distinct aromas, and flavors of citrus fruits. Among these compounds, limonene (**3**) is the most predominant in tangerine essential oil (EO), followed by γ‐terpinene (**2**), *p*‐cymene (**64**), α‐pinene (**5**), and myrcene (**8**) [10, 11, 12].

*C. reticulata* Blanco exhibits numerous biological activities such as antimicrobial, laxative, aphrodisiac, antiemetic, astringent, tonic, anticholesterolemic, anti‐inflammatory, expectorant, and hepatoprotective properties, widely used by the population for food consumption, both fresh and in various preparations (jams and sweets). The peel and fruit are employed in perfumery, aromatherapy, and cosmetics [10, 13].

Although numerous studies have highlighted the chemical composition of *C. reticulata* and its various biological activities, these data largely remain fragmented and concentrated in isolated experimental approaches, often lacking an integrated analysis that connects chemical composition with its pharmacological and therapeutic applications; however, this article proposes such a relationship. In addition, there is a shortage of studies exploring the influence of factors such as climate, soil, plant part used, and extraction methods on the variability of the identified bioactive compounds. Therefore, this review aims to analyze the main data available in the literature regarding the chemical composition and biological activities of *C. reticulata*, identifying patterns, potential applications, and areas that require further investigation.

The information in this review was obtained from the Science Direct, PubMed, and SciELO databases, covering the period from 2014 to 2024. Articles published in English or Portuguese were included, using the descriptors: chemical profile, biological activities, and *C. reticulata* Blanco. The search resulted in 1122 publications (716 in Science Direct, 388 in PubMed, and 18 in SciELO), which were subsequently analyzed according to the inclusion criteria: original articles with biological assays of extracts, EOs, fractions, or isolated compounds, as well as studies on chemical composition. Review articles, duplicates, publications outside the scope, or those that did not address biological activities were excluded. After screening, 49 articles met the established criteria. The systematic selection of these studies aims to consolidate a reliable scientific foundation for future research and sustainable applications of *C. reticulata* as a bioactive natural resource.

## 2 Results

### 2.1 Composition Chemical

The chemical investigation conducted on *C. reticulata* revealed the diversity of bioactive compounds distributed among the different fractions of the plant, ranging from the volatile constituents of the EO to complex nonvolatile secondary metabolites. The analysis allowed for the identification of monoterpenes and sesquiterpenes in the EO, including limonene, γ‐terpinene, α‐ and β‐pinene, sabinene, and α‐terpineol. These confer upon the species its characteristic aromatic profile, in addition to potential biological activities.

The nonvolatile extracts of the plant were demonstrated to contain substances of pharmacological and nutritional relevance, belonging to the classes of triterpenes, diterpenes, alkaloids, phenols, flavonoids, and coumarins. Among the flavonoids, methoxylated and glycosylated flavones and flavanones stand out, such as nobiletin, tangeretin, hesperidin, naringin, and quercetin, recognized for their antioxidant, anti‐inflammatory, and cardioprotective activities. Furthermore, coumarins and their derivatives were detected, substances with possible antimicrobial, antifungal, and hepatoprotective applications.

Other classes of compounds were also identified, including lipids (such as fatty acids and phytosterols), acridonic alkaloids, xanthine derivatives, tannins, depsides, as well as diverse constituents such as tocopherols, esters, and aliphatic alcohols. This complex chemical matrix highlights the multifunctional potential of *C. reticulata*, not only as a source of EO but also as a reservoir of active principles with therapeutic, cosmetic, food, and nutraceutical applications.

Thus, the following results are organized into chemical subgroups, in order to highlight the main constituents identified and their respective structural subclasses, reinforcing the phytochemical complexity of the species.

### 2.2 Volatile Components

From the gas chromatography–mass spectrometry (GC/MS) analysis of the EOs from *C. reticulata* fruit peels, it was possible to identify the presence of monoterpenes **1**–**13**. Monoterpenes **1**–**3** are unsaturated monocyclic compounds, and monoterpenes **4**–**7** are bicyclic with hydrocarbon or epoxide functions. Monoterpenes **4** and **6** showed similar structures, differing in the olefinic bond at C‐1, being endocyclic ∆^1,2^ and exocyclic ∆^1,7^, respectively [14, 15].

Monoterpenes **8**–**12** are acyclic compounds, with hydrocarbon, hydroxylated, or aldehydic functions, with one to three sp^2^ unsaturations. Compound **13** is a tricyclic sesquiterpenoid, with a 4/9/3 junction, featuring an oxirane as a functional group [14, 15]. In the investigations of Oliveira et al. [14], Sreepian et al. [15], and Kwangjai et al. [16], monoterpene **3** showed the highest chemical composition, with 85.7%, 72.53%, and 95.65% of the fruit peel EO, respectively.

In the analysis of the EO from *C. reticulata* fruit peels, carried out by Kwangjai et al. [16], the presence of monoterpenes **3**, **5**, **14**–**16** was identified and quantified. Compound **14** showed a cyclic structure with hydrocarbon function, with sp^2^ bonds. The bicyclic monoterpenes **5** and **15** showed structural similarity, differing in the site of the double bond, exhibiting endocyclic ∆^1,6^ and exocyclic ∆^1,10^ bonds, respectively. Monoterpene **16** showed a bicyclic structure, hydrocarbon function, and endocyclic sp^2^ unsaturation [15].

In a study carried out by Tao and Zhou [17], 22 terpenes and 5 aldehydes were elucidated by GC/MS‐FID, including acyclic, monocyclic, and bicyclic monoterpenes **1**, **3**–**5**, **7**–**9**, **14**–**25**, monocyclic sesquiterpenes **26**–**28**, diterpenoid **29**, and aldehydes **30**–**34**. Monoterpene **3** (60.74%) was evidenced as the major compound in the composition of the EO of ripe *C. reticulata* fruits. The bicyclic monoterpenes **4**, **5**, **15**–**17** showed the same degree of unsaturation (**3**), with methyl and/or isopropyl branching. Furthermore, monoterpenes **16** and **17** have the same composition and similar structures, differing in the position of the sp^2^ bond.

The acyclic monoterpenes **7**, **8**, and **18**–**24** are formed from hydrocarbons, primary or tertiary alcohol, or unsaturated monoaldehyde. In addition, monoterpenes **18** and **22** are geometric isomers, differing in spatial orientation. The monocyclic compounds **1**–**3**, **14**, **25**–**27** showed similar structures, differing in the degree of unsaturation, position of the sp^2^ bond, functional group, and number of substituents. Furthermore, sesquiterpenes **26** and **27** resemble each other, differing in the position of the double bond, alternating between exocyclic or endocyclic [17].

Sesquiterpene **28** showed a 10‐carbon ring, 3 sp^2^ unsaturations, and dimethyl and isopropyl branching [15, 17]. Diterpenoid **29** was the most complex constituent, presenting an acidic portion (–SO_3_H), two basic portions (–N–), and four saline portions (–CO_2_Na), in addition to presenting polysubstituted benzene rings [17].

Aldehydes **30**–**34** showed simple structures with 8–12 saturated carbons, and aldehyde **35** showed an unsaturated 10‐carbon chain [17, 18, 19, 20, 21].

In the study carried out by Lin et al. [21], the metabolic composition of EOs from the fruit peels of two *Citrus* species, *C. reticulata* Blanco cv. Kinokuni (OECrBK) and *C. reticulata* Blanco (OECrB), was investigated. Even though they are of the same species, they showed some divergences in compounds and composition. A total of 35 compounds were identified, corresponding to the following terpenes **1**–**8**, **10**, **13**, **15**, **19**, **23**, **24**, **26**, **27**, and **36**–**53**, representing 98.68% (OECrBK) and 99.42% (OECrBK) of the EO composition.

Approximately 97% of the compounds are monoterpenes, and about 2% correspond to sesquiterpenoids. Monoterpene hydrocarbons were in greater abundance (93.96% and 95.94%, respectively), whereas oxygenated monoterpenes (2.88% and 0.95%, in that order) were less expressive [21].

In both EOs, monoterpene **3** was observed as the major component, corresponding to 79.13% (OECrBK) and 86.03% (OECrB). Regarding the structures of terpenes and aldehydes, compounds **1**–**35** were previously reported. Sesquiterpene **36** exhibits an acyclic structure, with an unsaturated hydrocarbon function. The acyclic monoterpene **37** showed a similar structure to **18**, differing in the oxygenated functional group.

The monocyclic terpenes **38**–**41** showed the same structural skeleton, differing in the position of the sp^2^ bonds, branching, and functional group, varying between hydrocarbon and aldehydic. Sesquiterpene **40** showed structural similarity to sesquiterpene **27**, differing in an exocyclic double bond at ∆^12,13^, present in compound **40**. Sesquiterpenes **42**–**48** have similar structures, with two or three unsaturated hydrocarbon rings, except for sesquiterpene **45**, which presented a hydroxyl [15, 21].

According to the analysis of the EO from fresh *C. reticulata* Blanco cv. Dancy fruit peels, it was possible to identify terpenes **1**, **3**, **5**, **8**, **11**, **12**, **15**, **18**, **19**, **23**–**25**, and **36**–**38**, previously characterized, as well as identifying monoterpene **3** (94.59%) as the main constituent of *C. reticulata* EO [18].

Compound **49**, identified in OECrB, exhibited a different structure from the others, presenting an 11‐membered ring with 3 sp^2^ unsaturations. Monoterpenoid **50**, identified in OECrBK oil, exhibited a differentiated structure as it is a monophenol. Sesquiterpenes **51**–**53** are monocyclic and bicyclic structures, with hydrocarbon or oxirane organic functions [21].

Eighteen terpenes of varied structures were identified from the EO of *C. reticulata* fruits, such as terpenes **1**, **3**–**6**, **8**, **19**, **24**, **36**, **39**, **42**, and **43**, previously mentioned, along with compounds **54**–**58**. The monocyclic monoterpenes **54**–**56** are structurally similar, differing in the position of the sp^2^ unsaturation, varying between endocyclic and exocyclic, functional group, and degrees of unsaturation. Sesquiterpenes **57** and **58** are unsaturated cyclic hydrocarbon compounds. Furthermore, compound **57** is similar to **41**, differing in the sp^2^ bond, varying between endocyclic and exocyclic. Monoterpene **3** (80.2%) was evidenced as the major constituent of the EO [19].

In the characterization analysis of the constituents of the EO from *C. reticulata* peel residues, monoterpenes **3**, **5**, **37**, and **41**, previously mentioned, along with monoterpenes **59**–**63,** were identified. The monocyclic monoterpene **59** is structurally similar to monoterpenes **2** and **14**, differing in the sp^2^ olefinic bonds [20].

The acyclic monoterpene **60** showed structural similarity to compounds **7** and **8**, distinguishing them by the location of one or two sp^2^ bonds, varying between endocyclic and exocyclic. Monoterpene **61** is similar to monoterpene **19**, differing in the functional group; compound **61** has an apolar character due to the presence of ethanoate, whereas substance **19** has a polar character due to the presence of hydroxyl [20].

Substance **62** is similar to monoterpenes **16** and **17**, except for the position of the sp^2^ unsaturated bond, which is located at C‐4, C‐3, and C‐2, respectively. Monoterpene **63** exhibited a saturated cyclohexane, interconnected to cyclopropane at C‐3 and C‐6, with three methyl branches and one endocyclic sp^2^ unsaturated bond [20].

In the study of characterization of volatile metabolites present in the EO of fresh *C. reticulata* peels, subclasses of terpenes of varied structures were identified, including monoterpenes **1**–**7**, **9**, **11**, **15**, **16**, **18**–**21**, **23**–**25**, **38**, **39**, **41**, **50**, **53**, **60**, and **63**, and sesquiterpenes **26**–**28**, **52**, and **57**, previously described, along with monoterpenes **64**–**75**.

Monoterpenes **64**–**73** exhibited monocyclic or bicyclic unsaturated structures or an aromatic ring, with branching and hydrocarbon or oxygenated function. Monoterpenes **67** and **68** are geometric isomers, exhibiting a monounsaturated ring, with isopropyl substitution for hydroxyl. Monoterpene **74** is a geometric isomer of compound **7**, whereas monoterpene **75** exhibits similarity to substance **60**, differing in the presence of the carbonyl at C‐3 [22].

Terpenes **2**, **3**, **10**, **21**, **25**, **27**, **28**, **37**, **40**, **50**, **52**, **63**, and **76**–**78** were evidenced in the EO of *C. reticulata* Blanco and *C. reticulata* Chachi peels, with monoterpene **3** (88.4% and 75.1%, respectively) as the main constituent. Monoterpenoid **76** and sesquiterpenes **77** and **78** are low‐polarity terpenes, presenting acyclic or cyclic structures, with hydrocarbon or oxygenated function [23].

### 2.3 Nonvolatile Compounds

#### 2.3.1 Terpenes

From the hexane extract of *C. reticulata* stem bark, it was possible to identify two polycyclic triterpenoids, compounds **79** and **80**. Both had a secondary hydroxyl, exhibiting a 6/6/6/6/5‐membered ring (**79**) and a 6/6/6/6/6‐membered ring (**80**) [24].

In a study conducted by Phetkul et al. [25], two polycyclic triterpenoids, **81** and **82**, were purified from dichloromethane (CH_2_Cl_2_) and acetone (Me_2_CO) extracts. Triterpenoid **81** exhibited a unique six‐ring structure with a 6/5/6/6/6 junction, and the sixth ring being a 1,4‐epoxybut‐di‐ene branch. The main chain consisted of lactones, epoxides, and a ketone. Triterpenoid **82** had five rings, a 6/6/6/6/5 junction, with alcoholic and acidic organic functions.

The acyclic triterpene **83** and diterpenoids **84** and **85** were identified in the gradient solvent extraction (petroleum ether, chloroform, ethyl acetate [EtOAc], carbinol, and aqueous) of *C. reticulata* fruit peels. Triterpene **83** had an acyclic carbon chain, diterpenoid **84** consisted of five rings, a 6/6/6/5/4 junction, and two oxygenated groups, whereas diterpenoid **85** exhibited an acyclic chain containing an olefinic sp^2^ (Srimathi and Gurunathan, 2020) [26]. These terpenes were the highest molecular weight constituents of the terpenic class found in this study.

#### 2.3.2 Coumarins

According to the purification of the EtOAc:hexane fraction from *C. reticulata* stem bark, coumarins **86** and **87** were evidenced. Coumarins have a defined structural skeleton, consisting of an aromatic ring linked to an unsaturated ∆^3,4^ lactone. Coumarin **86** had methoxyls at C‐6 and C‐7, and coumarin 87 had a dimethyl pyrone [24].

Seven coumarins, **88**–**94**, were identified and purified from CH_2_Cl_2_ and Me_2_CO extracts. Coumarins **88** and **89** have similar structures, differing in the oxygenated functional group. Coumarin **90** presented methoxyl, hydroxyl, and glutamic acid linked to an oxygenated group. Meanwhile, coumarins **91**–**94** showed mixed structures, with functions varying between hydroxylated, ether, and ester [25].

#### 2.3.3 Flavonoids

Diverse flavonoids are identified from extracts of different solvents of dried leaves, fresh fruit peels, pulp, and seeds of *C. reticulata*. Eighty‐eight compounds of this class were identified in solvents of different polarities.

From the fractionation of the EtOAc extract of *C. reticulata* Blanco fruit peels, it was possible to identify flavonoids **95**–**100**. These flavonoids had a polymethoxylated skeleton. Flavones **95**–**97** showed structural similarity, differing at C‐6 and C‐8, where they presented differences in functional position and number of methoxyls. Flavonols **98** and **99** have a methoxyl linked to C‐3 and polymethoxyl groups throughout the chain. Flavonol **100** stood out for presenting a C‐3‐*O*‐β‐glucose portion and another 3‐hydroxy‐3‐methyl‐5‐methoxy‐glutaroyl portion [27].

Seven flavonoids were identified from the fractionation of ethanolic (70%) and water extracts of fresh *C. reticulata* Blanco fruit peels. Flavanone‐*O‐*glycosides **101** and **102**, flavone*‐O*‐glycoside **103**, flavanones **104** and **105**, flavonol **106**, and flavone‐*O*‐methoxylated **107** were identified. These flavonoids have polyhydroxyls and methoxyls as functional groups [28].

The purification of the fractionation of the methanolic extract of dried *C. reticulata* leaves allowed the identification of 15 flavonoids of varied subclasses, such as flavones **97** and **107**–**111**, flavonols **112**–**115**, flavanones **116**–**119**, and methoxylated chalcone **120**, presenting polyhydroxyls or polymethoxylated groups [26, 29].

According to a study conducted by Wang et al. [1], seven flavonoids were identified from the acetone extracts of the peels, pulps, and seeds of *C. reticulata* Blanco cv Chachiensis. Thus, flavone‐*O*‐methoxylated **95** and **107**, flavanone‐*O*‐glycosides **101**, **102**, and **121**, flavone‐*O*‐glycosides **103**, and flavanones **105** were observed, with flavanone **101** (7497.15 µg g^−1^) as the major compound. Flavones **95**, **97**, **107**, and **119** were also evidenced in the ethanolic (95%) extract of *C. reticulata* peels, with flavone **107** (33.87%) observed as the major compound, followed by flavone **95** (20.98%). These flavones presented polysubstitutions, with polymethoxyls, hydroxyls, or both oxygenated groups [30].

Flavones **95**, **97**, and **107** and flavanones **101**, **102**, **104**, and **121** were also reported in methanolic (80%) extracts of *C. reticulata* peels from 12 genotypes, along with flavan‐3‐ol **122**, flavanone‐*O*‐glycosides **123**, **124**, and **125**, flavones **126**–**128**, flavonol **129**, flavanones **130** and **131**, and flavone‐*O*‐glycoside **132** and **133**, with compound **100** (varying 17.53–55.98 mg g^−1^ DW) evidenced as the major flavonoid in this extract. These flavonoids presented polysubstitutions, exhibiting different branching, such as glycosides, ketones, hydroxyls, and methoxyls [31].

Some of these compounds, **95**, **97**, **101**, **102**, **121**–**126**, **130**, and **132**, were identified in at least 1 of the 12 genotypes of the methanolic (80%) extract of *C. reticulata* pulp, with compound **101** (6.721–22.13 mg g^−1^ DW) as the major compound of this class [32].

Flavanone‐*O*‐glycosides **101**, **124**, and **130** were also identified in immature fruits of different sizes (8–24 mm) of *C. reticulata*, as well as flavone‐*O*‐glycoside **133**. Flavanone **101** was observed with the highest content, decreasing as the fruit size increased [33].

From the polymethoxyflavonoid‐enriched fractionation of the ethanolic (95%) extract of *C. reticulata* Chachi fruit peels, it was possible to identify and purify flavones **95** and **98**, previously reported in EtOAc extracts, along with flavones **134**, **135**, **136**, and **137** and flavanones **138** and **139**. Flavones **134** and **135** show structural and functional similarity, distinguished by the presence of a methoxyl at C‐3′ in compound **134**. Methoxylated flavonol **98** and flavones **136** and **137** resemble the major compound **95** (49.28%), differing in the methoxyl binding site. Flavanones **138** and **139** exhibit similar structures and functions, differing in the position of a methoxyl [34, 35].

In the study by Wang et al., [1] the metabolic composition of the ethanolic (95%) extract of *C. reticulata* fruit peels was evaluated, and flavonoids **101**, **102**, **121**, **123**–**125**, **130**, **132**, and **135**, previously observed in methanolic (80%) extracts of *C. reticulata* peels, as well as flavonoids **140**–**150**, were identified. Flavones **140**–**142** are similar, exhibiting polymethoxylated groups with a hydroxyl group, differing in the binding site of the functional groups.

Flavones **142** and **143** have polyhydroxyls and glycoside groups, whereas flavone **144** presented a very differentiated structure; in addition to the glycoside linked to C‐7, it exhibited seven silicons (Si) along the chain. Flavanones **145** and **146** presented similar structures, distinguishing by the branching of the glycoside and the presence of hydroxyl at C‐3′ in compound **146**. Flavanone **147** presented methoxylated groups in cycles A and B, and flavones **148**–**150** showed some similarities, differing in the number of methoxylated groups and absence of hydroxyl in compound **150** [1].

Sixteen flavonoids were purified in studies conducted by Phetkul et al. [25], including flavonoids **95**, **101**, **103**, **107**, **109**, **111**, **113**, **114**, **119**, **120**, **123**, **124**, **136**, **139**, and **142**, detailed previously, along with flavanone **151**. Flavanone **151** is similar to flavone **148**, differing in the presence of sp^2^ unsaturation at C‐2, exhibited by compound **148** [35].

In the investigation carried out by Costanzo et al. [36, 37], flavonoids **97**, **102**, **103**, **105**, **106**, **126**, and **128**, previously mentioned, as well as compounds **152**–**179**, were identified in aqueous/methanol (8:2) and methanol extracts of *C. reticulata* peels, pulp, and seeds. Anthocyanidins **152**–**164** are polyhydroxylated compounds with glycoside groups, whereas anthocyanidins **165** and **166** presented only hydroxyls. Flavan‐3‐ols **167** and **168** differ in the binding site of the phenol acid [25].

Flavanone **169** exhibited a similar framework to **144**, distinguishing by the binding site of the trimethylsilyloxyphenyl cycle and the number of silicon atoms [36, 37]. Compounds **170**–**175** are methoxylated flavonols, with one or two glycoside groups, polyhydroxylated. Flavones **176**–**178** presented, in addition to glycoside groups, hydroxylated or methoxylated functions, differing in the number of functional groups and spatial orientation. Isoflavone **179** exhibited hydroxylated functions and a glycoside linked to C‐8 [25].

In studies conducted by Apraj and Pandite [38], metabolites from the alcoholic extract of *C. reticulata* peel skin were characterized by hot air (Soxhlet) and cold air (maceration) extraction methods, with methoxylated flavonol **180** and flavones **181**–**183** being observed. Metabolites **180** and **183** are polymethoxylated, both showing similar structures, distinguished by the binding site and organic function, varying between hydroxyl and methoxyls.

Some flavonoids were identified in more than one type of extract, such as acetone, methanol, and ethanol, in different parts of the fruit, such as peels, pulp, seeds, and whole fruit, evidencing the richness of this class of compounds throughout the fruit.

#### 2.3.4 Phenolic Acids and Diverse Compounds

In the study conducted by Ferreira et al. [28], different classes of compounds were isolated; in addition to the previously mentioned flavonoids, phenolic acids **184**–**186** were also identified. Phenolic acids **184** and **185** have similar structures, differing in the presence of hydroxyl or methoxyl at C‐3, respectively. Phenol **186** exhibited a mixed chain of polar character due to the presence of polyhydroxyls. These phenols were also identified in acetone extracts of *C. reticulata* Blanco cv Chachiensis peels, pulps, and seeds, along with phenolic acid **187**. Phenol **187** is analogous to **184** and **185**, differing by the absence of an oxygenated group at C‐3 [39].

Phenolic acids **184**–**187** were also reported in 12 genotypes of methanolic (80%) extracts of wild *C. reticulata* fruit peels and pulp, as well as phenols **188**–**191**. Monophenol acid **188** presented four aromatic, methoxylated, propenoic, and hydroxylated substitutions, whereas phenols **189**–**191** exhibited simple structures, with a substituted aromatic ring, classified as biphenol acid, monophenol acid, and methoxylated monophenol acid, respectively. Compound **185** (1730.93–13 607.19 µg g^−1^ DW and 778.06–2336.07 µg g^−1^ DW, respectively) was the predominant compound in the 12 genotypes [31, 32].

In a study conducted by Phetkul et al. [25], previously mentioned, phenolic acids **189** and **190**, hydroxybenzaldehyde **192**, phenolic methanoate **193**, and depsides **194** and **195** were identified and purified. Phenols **189**, **190**, and **192** presented similar and basic structures, consisting of an aromatic ring, differing in the number of hydroxyls and functional group. Phenol **193** has a mixed chain, with an aromatic ring acyclic portion, with different oxygenated functions. Depsides **194** and **195** are composed of two aromatic units interconnected by an ester, in addition to presenting varied oxygenated functional groups.

The work of Costanzo et al. [36, 37] identified phenols of varied subclasses, such as acid **186**, previously described, along with acids **196**–**198** and **199**, benzoate **200**, polyphenols **201** and **202**, and benzaldehyde **203**. Phenolic acids **196** and **197** are simple compounds with similar structures and functional groups at the same binding sites, differing in organic function.

Tannin **198** presented a portion similar to acid **196**, linked to the tannin group. Acid **199** presented a mixed chain, with interconnected rings by a methyl propenoate portion. Benzoate **200** exhibits the same structural skeleton as acid **196**, distinguishing in the functional group. Polyphenols **201** and **202** are 1‐propranones linked to two phenols; the latter compound also exhibited a glycoside. Benzaldehyde **203** is structurally similar to benzoate **200**, discerning in the presence of methoxyls in *para* substitutions and in the priority functional group [36, 37].

Phenol **204** was identified in the EO of fresh *C. reticulata* fruit peels. This phenol presented a basic structure, with methanoate interconnected to the aromatic ring, showing an *ortho* relationship with the hydroxyl group [22].

#### 2.3.5 Alkaloids

In the study by Ye et al. [29], a pyrano[2,3‐a]acridone‐type alkaloid **205** was observed for the first time in an MeOH extract of the *Citrus* genus, in addition to nine other known acridonic alkaloids **206**–**209**, **210**–**214**, and aldehyde **215**. These alkaloids presented 6/6/6/6 or 6/6/6 polysubstituted junctions. Alkaloid **205** showed similarity to **206**, differing in the linear pyrano[2,3‐a]acridone **205**, whereas alkaloid **206** exhibited a pyrano[2,3‐c]acridone portion.

Alkaloids **206**–**209** presented the same structural skeleton, differing at C‐5 and C‐6 by their functional groups, exhibiting hydroxyls and/or methoxyls. Compounds **210**–**214** exhibited the same skeleton, differing in functional groups at C‐4, C‐6, C‐9, and C‐10, presenting methoxyls and/or hydroxyls. Xanthine **215** is a nitrogenous base, with a pyrimidine ring interconnected to an imidazole [29].

Acridonic alkaloids **207**–**209** were also reported in CH_2_Cl_2_ and Me_2_CO extracts of *C. reticulata* Blanco peels, leaves, and branch barks, as well as acridonic alkaloids **216** and **217**. These alkaloids presented a 6/6/6 structural configuration, with two aromatic rings, one at each end, and a central saturated cycle, differentiating them by their functional groups [25].

#### 2.3.6 Lipids

In the study by Tahsin et al. [24], previously mentioned, in addition to terpenes and coumarins, the identification of two phytosterols **218** and **219**, and one fatty acid **220** was also possible. Phytosterols **218** and **219** have the same structural skeleton, corresponding to a mixed chain, with a fused four‐ring framework, a 6/6/6/5 junction, with dimethyl branching, an acyclic group, and an oxygenated group. They differ in the unsaturation present in compound **218**. Fatty acid **220** is a common, saturated 16‐carbon carboxylic acid [38].

From the gradient extract, carried out by Srimathi and Gurunathan [26], fatty acid **221**, sterol **222**, and cholesterol **223** were identified. Fatty acid **221** presented a structure with 20 carbons, acyclic, and saturated. Sterol **222** is similar to **219**, differing in the oxygenated group. Cholesterol **223** resembles sterol **218**, differing in an sp^2^ unsaturation and absence of ethyl in the acyclic portion [37].

#### 2.3.7 Diverse Classes

An active form of vitamin E **224** was among the variety of metabolites identified from *C. reticulata* fruit peels. This compound **224** consists of a monophenol, a pentahydrofuran ether, and a terpenic portion [26].

Diverse compounds of varied classes were identified in the work of Phetkul et al. [25]; among these, polymethoxylated benzene **225** was purified, consisting of an aromatic ring with varied oxygenated and hydrocarbon functional groups.

Aldehydes **32**, **34**, **35**, and **33**, previously reported, hydrocarbon **226**, esters **227** and **228**, and carboxylic acid **229**. Hydrocarbon **226** contains 20 carbons and an sp‐type unsaturation at carbons ∆^9,10^ [38]. Esters **227** and **228** are compounds of 5 and 18 carbons, respectively, with distinct oxygenated functional groups. Carboxylic acid **229** exhibited an 18‐carbon structure with 2 unsaturations [25, 26].

In the work carried out by Apraj and Pandite [38], in addition to terpenes and flavonoids, various organic metabolites were identified, such as carboxylic acids **229** and **234**, esters **230**–**232** and **110**, organosulfur acid **233**, pyran **235**, aldehyde **236**, alcohol **237**, amide **108**, hydrocarbon **109**, and ketone **111**.

Compounds **229**–**232**, **234**, **239**, and **240** presented simple acyclic structures, differing in the number of carbons, functional groups, type of C–C bond, and branching. Acid **233** exhibits a relatively low molecular weight structure, with ketone functions and a sulfhydryl group (HS–). Compounds **235**–**237** presented monocyclic structures, varying between five and six members and different oxygenated functional groups.

Amide **238** presented three nitrogenous groups interconnected to the carbon portion. Ketone **241** is a complex structure, presenting two aromatic rings and two six‐membered rings, a 6/6–6/6 ring junction at each end, interconnected by an endocyclic bond and methoxyl functions [38].

In the research by Bhandari et al. [22], in addition to terpenes, aldehydes **30**–**34**, previously described, hydrocarbon **242**, and alcohols **243** and **244** were also identified. Hydrocarbon **242** consists of two aromatic rings, whereas alcohols **243** and **244** exhibit open, saturated chains with eight or ten carbons (Table 1).

**TABLE 1:** Chemical profile of *Citrus reticulata* Blanco ordered by classes.

Parts of the plant	Subclass	Compounds	S. no
Compounds identified in the essential oil of *Citrus reticulata* (volatile)			
Fruit peels	Monocyclic monoterpenoid	α‐Terpineol	1
Fruit peels	Bicyclic monoterpenoid	γ‐Terpinene	2
Fruit peels	Monocyclic monoterpenoid	Limonene	3
Fruit peels	Bicyclic monoterpenoid	α‐Thujene	4
Fruit peels	Bicyclic monoterpenoid	α‐Pinene	5
Fruit peels	Bicyclic monoterpenoid	Sabinene	6
Fruit peels	Acyclic monoterpene	*trans*‐Limonene oxide	7
Fruit peels	Acyclic monoterpene	β‐Myrcene	8
Fruit peels	Monocyclic monoterpene	*E*‐Ocimene	9
Fruit peels	Acyclic monoterpene	Citronellal	10
Fruit peels	Acyclic monoterpene	Geraniol	11
Fruit peels	Acyclic monoterpene	Geranial	12
Fruit peels	Tricyclic sesquiterpenoid	Caryophyllene oxide	13
Fruit peels	Monocyclic monoterpene	β‐Phellandrene	14
Fruit peels	Bicyclic monoterpene	β‐Pinene	15
Fruit peels	Bicyclic monoterpene	*δ*‐3‐Carene	16
Fruit peels	Bicyclic monoterpene	2‐Carene	17
Fruit peels	Monocyclic monoterpene	β‐Citral	18
Fruit peels	Acyclic monoterpenoid	Linalol	19
Fruit peels	Acyclic monoterpenoid	β‐Citronellal	20
Fruit peels	Monocyclic monoterpenoid	β‐Citronellol	21
Fruit peels	Acyclic monoterpenoid	α‐Citral	22
Fruit peels	Acyclic monoterpenoid	Terpinolene	23
Fruit peels	Acyclic monoterpenoid	Terpinen‐4‐ol	24
Fruit peels	Monocyclic monoterpenoid	Perillaldehyde	25
Fruit peels	Thymol diterpenoid	*δ*‐Elemene	26
Fruit peels	Monocyclic sesquiterpene	γ‐Elemene	27
Fruit peels	Monocyclic sesquiterpene	Germacrene D	28
Fruit peels	Monocyclic sesquiterpene	Methylthymol	29
Fruit peels	Aldehyde	Octanal	30
Fruit peels	Aldehyde	Nonanal	31
Fruit peels	Aldehyde	Decanal	32
Fruit peels	Aldehyde	Undecanal	33
Fruit peels	Aldehyde	Dodecanal	34
Fruit peels	Aldehyde	*E*‐2‐Decenal	35
Fruit peels	Acyclic sesquiterpene	α‐Farnesene	36
Fruit peels	Acyclic monoterpenoid	Neryl acetate	37
Fruit peels	Monocyclic monoterpenoid	Carvone	38
Fruit peels	Monocyclic monoterpene	α‐Terpinene	39
Fruit peels	Bicyclic sesquiterpene	β‐Elemene	40
Fruit peels	Bicyclic sesquiterpene	α‐Phellandrene	41
Fruit peels	Monocyclic sesquiterpene	α‐Copaene	42
Fruit peels	Monocyclic sesquiterpene	*δ*‐Cadinene	43
Fruit peels	Monocyclic monoterpenoid	β‐Selinene	44
Fruit peels	Bicyclic sesquiterpenoids	Cubenol	45
Fruit peels	Bicyclic sesquiterpenoids	β‐Eudesmol	46
Fruit peels	Monocyclic monoterpene	Valencene	47
Fruit peels	Bicyclic monoterpenoid	α‐Selinene	48
Fruit peels	Bicyclic sesquiterpene	α‐Humulene	49
Fruit peels	Bicyclic sesquiterpene	Thymol	50
Fruit peels	Bicyclic sesquiterpene	*E*‐Caryophyllene	51
Fruit peels	Monocyclic sesquiterpene	Germacrene B	52
Fruit peels	Bicyclic sesquiterpene	*cis*‐Limonene Oxide	53
Fruit peels	Monocyclic monoterpenoid	*Iso‐*menthone	54
Fruit peels	Monocyclic monoterpene	*p*‐Mentha‐1(7),8‐diene	55
Fruit peels	Monocyclic monoterpene	*p*‐Mentha‐2,4(8) diene	56
Fruit peels	Bicyclic sesquiterpene	γ‐Muurolene	57
Fruit peels	Monocyclic sesquiterpene	Β‐Bisabolene	58
Peel residues	Monocyclic monoterpene	Cyclohexene,4‐methylene‐1‐(1‐methylethyl)‐	59
Peel residues	Acyclic monoterpene	α‐Myrcene	60
Peel residues	Acyclic monoterpenoid	Linalyl acetate	61
Peel residues	Bicyclic monoterpene	4‐Carene	62
Peel residues	Bicyclic monoterpene	Camphene	63
Fruit peels	Monocyclic monoterpene	Cymene	64
Fruit peels	Monocyclic monoterpene	*p*‐Cymenene	65
Fruit peels	Monocyclic monoterpenoid	1,8‐Cineole	66
Fruit peels	Monocyclic monoterpenoid	*Trans*‐*p*‐Mentha‐2,8‐dien‐1‐ol	67
Fruit peels	Monocyclic monoterpenoid	*Cis*‐*p*‐mentha‐2,8‐dien‐1‐ol	68
Fruit peels	Monocyclic monoterpenoid	Geijerene	69
Fruit peels	Monocyclic monoterpenoid	1,8‐Menthadien‐4‐ol	70
Fruit peels	Monocyclic monoterpene	Thymol methyl ether	71
Fruit peels	Monocyclic monoterpenoid	*Trans*‐Carveol	72
Fruit peels	Bicyclic monoterpenoid	Camphor	73
Fruit peels	Acyclic monoterpene	*Z*‐Ocimene	74
Fruit peels	Acyclic monoterpenoid	2‐Methyl‐6‐methylen‐octa‐1,7‐dien‐3‐one	75
Fruit peels	Acyclic monoterpenoid	Geranyl acetate	76
Fruit peels	Monocyclic sesquiterpenoid	Cyclohexane,2,4‐diisopropenyl‐1‐methyl‐1‐vinyl	77
Fruit peels	Tricyclic sesquiterpenoid	(−)‐Spathulenol	78
	**Compounds identified from extracts of *C. reticulata***		
Stem bark	Polycyclic triterpenoid	Lupeol	79
Stem bark	Polycyclic triterpenoid	β‐amyrin	80
Fruit peels, twig bark, and leaves	Polycyclic triterpenoid	Limonin	81
Fruit peels, twig bark, and leaves	Polycyclic triterpenoid	Betulinic acid	82
Fruit peels	Acyclic triterpene	Squalene	83
Fruit peels	Polycyclic diterpenoid	Spiro[androst‐5‐ene‐17,1′‐cyclobutan]‐2′‐one,3‐hydroxy‐[3beta‐17beta]	84
Fruit peels	Acyclic diterpenoid	3,7,11,15‐Tetramethyl‐2‐hexadecene‐1‐ol	85
**Coumarin**			
Stem bark	Coumarin	Scoparone	86
Stem bark	Pyranocoumarin	Xanthyletin	87
Stem bark	Coumarin	Scopoletin	88
Stem bark	Glycoside	Crenulatin	89
Stem bark	Coumarin	8,3′‐β‐Glucosyloxy‐2′‐hydroxy‐3′‐methylbutyl‐7‐methoxy‐coumarin	90
Stem bark	Coumarin	5‐(2‐Enyl‐3‐methylbut)oxy‐7‐hydroxycoumarin	91
Stem bark	Coumarin	7‐Geranyloxy coumarin	92
Stem bark	Coumarin	6,8‐Dimethoxypentylisocoumarin	93
Stem bark	Coumarin	8‐Hydroxy‐6‐methoxy‐pentylisocoumarin	94
**Flavonoids**			
Fruit peels	*O*‐methoxylated flavone	Nobiletin (5,6,7,8,3′,4′‐hexamethoxyflavone)	95
Fruit peels	Flavone	Isosinensetin	96
Fruit peels	Methoxylated flavone	Sinensetin (5,6,7,3′,4′‐pentamethoxyflavone)	97
Fruit peels	Methoxylated flavonol	3,5,6,7,8,3′,4′‐Heptamethoxyflavone	98
Fruit peels	Flavonol	3,5,7,8,2′,5′‐Hexamethoxyflavone	99
Fruit peels	Flavone glycoside	Citrusunshitin A	100
Fruit peels	Flavanone‐*O*‐glycosides	Hesperidin	101
Fruit peels	Flavanone‐*O*‐glycosides	Naringin	102
Fruit peels	Flavone‐*O*‐glycosides	Rutin	103
Fruit peels	Flavanone	Naringenin	104
Fruit peels	Flavonone	Hesperetin	105
Fruit peels	Flavonol	Quercetin	106
Fruit peels	*O*‐Methoxylated‐flavone	Tangeretin	107
Fruit peels	Flavone	5,6,4′‐Trihydroxypyranoflavone	108
Fruit peels	Flavonol	Nobletin	109
Fruit peels	Flavanone	5‐Hydroxy‐4′,6,7,8‐tetramethoxyflavone	110
Fruit peels	Flavanone	5‐Demethylnobiletin	111
Fruit peels	Methoxylated chalcone	Citrusinol	112
Fruit peels	Methoxylated flavone	3‐Hydroxytangeretin	113
Fruit peels	Flavonol	Natsudaidai	114
Fruit peels	Flavonol	Sudachitin	115
Fruit peels	Flavonone	4′‐Hydroxyisolonchocarpin	116
Fruit peels	Flavonone	Citflavanone	117
Fruit peels	Methoxylated flavone	5‐Hydroxy‐6,7,8,3′,4′‐pentamethoxyflavanone	118
Fruit peels	*O*‐Methoxylated flavone	5‐Hydroxy‐6,7,8,4′‐tetramethoxyflavanone	119
Fruit peels	Flavonol	Chalcone	120
Fruit peels	Flavanone *O*‐glycosides	Neohesperidin	121
Fruit peels	Flavan‐3‐ol	Taxifolin	122
Fruit peels	Flavanone‐*O*‐glycoside	Eriocitrin	123
Fruit peels	Flavanone‐*O*‐glycoside	Narirutin	124
Fruit peels	Flavanone	Poncirin	125
Fruit peels	Methoxylated flavonol	Quercitrin	126
Fruit peels	Flavone	Luteolin	127
Fruit peels	Flavone	Diosmetin	128
Fruit peels	Flavonol	Kaempherol	129
Fruit peels	Flavanone‐*O*‐glycosides	Didymin	130
Fruit peels	Flavanone glycoside	Eridictyol	131
Fruit peels	Flavone *O*‐glycoside	Rhoifolin	132
Fruit peels	Flavone‐*O*‐glycosides	Diosmin	133
Fruit peels	Flavone	5‐Hydroxy‐6,7,8,3′,4′‐pentamethoxyflavone	134
Fruit peels	Flavone	5‐Hydroxy‐6,7,8,4′‐tetramethoxyflavone (gardenin B)	135
Fruit peels	Flavone	5,6,7,8,4′‐Pentamethoxyflavone (tageretin)	136
Fruit peels	Flavone	5,6,7,3′,4′‐Pentamethoxyflavone	137
Fruit peels	Flavanone	6,7,8,3′,4′‐Pentamethoxyflavanone	138
Fruit peels	Flavanone	5,6,7,3′,4′‐Pentamethoxyflavanone	139
Fruit peels	Flavone	6‐*O*‐Desmethyl tangeritin	140
Fruit peels	Flavone	7‐*O*‐Desmethyl tangeritin	141
Fruit peels	Flavone C‐glucoside	6,8‐di‐C‐β‐d‐Glucosilapigenina (vicenin‐2)	142
Fruit peels	Flavone C‐glucoside	Diosmetin‐6‐C‐glucoside	143
Fruit peels	Flavone C‐glucoside	Apigenin‐8‐C‐glusoside	144
Fruit peels	Flavanone *O*‐glycoside	Neoeriocitrin	145
Fruit peels	Flavanone glycoside	Melitidin	146
Fruit peels	Flavanone	Naringenin trimethyl ether	147
Fruit peels	Flavone	5‐Hydroxy‐7,4′‐dimethoxyflavone	148
Fruit peels	Flavone	Tymusin	149
Fruit peels	Flavone	5,7,8,4′‐Tetramethyl‐*O*‐isocutellarein	150
Fruit peels	Flavanone	2,3‐Dihydro‐5‐hydroxy‐4′,7‐dimethoxy‐flavanone	151
Fruit peels, pulp, and seeds	Anthocyanidin glucoside	Delphinidin‐3,5‐diglucoside	152
Fruit peels, pulp, and seeds	Anthocyanidin glucoside	Cyanidin‐3,5‐di‐*O*‐glucoside	153
Fruit peels, pulp, and seeds	Anthocyanidin glucoside	Delphinidin‐3‐*O*‐glucoside	154
Fruit peels, pulp, and seeds	Anthocyanidin glucoside	Cyanidin‐3‐*O*‐glucoside	155
Fruit peels, pulp, and seeds	Anthocyanidin glucoside	Delphinidin‐3‐*O*‐arabinoside	156
Fruit peels, pulp, and seeds	Anthocyanidin glucoside	Petunidin‐3‐*O*‐glucoside	157
Fruit peels, pulp, and seeds	Anthocyanidin glucoside	Cyanidin‐3‐*O*‐arabinoside	158
Fruit peels, pulp, and seeds	Anthocyanidin glucoside	Pelargonidin‐3‐*O*‐glucoside	159
Fruit peels, pulp, and seeds	Anthocyanidin glucoside	Peonidin‐3‐*O*‐glucoside	160
Fruit peels, pulp, and seeds	Anthocyanidin glucoside	Malvidin‐3‐*O*‐glucoside	161
Fruit peels, pulp, and seeds	Anthocyanidin glucoside	Malvidin‐3‐*O*‐arabinoside	162
Fruit peels, pulp, and seeds	Anthocyanidin glucoside	Delphinidin rutinoside	163
Fruit peels, pulp, and seeds	Anthocyanidin glucoside	Malvidin 3‐*O*‐*p*‐coumaroylglucoside	164
Fruit peels, pulp, and seeds	Anthocyanidin	Delphinidin	165
Fruit peels, pulp, and seeds	Anthocyanidin	Pelargonidin	166
Fruit peels, pulp, and seeds	Flavan‐3‐ol	Epigallocatechin‐3‐gallate	167
Fruit peels, pulp, and seeds	Flavan‐3‐ol	Gallocatechin‐3‐gallate	168
Fruit peels, pulp, and seeds	Flavanone glycoside	Naringenin‐7‐*O*‐neohesperidoside	169
Fruit peels, pulp, and seeds	Flavonol glucoside	Myricitrin	170
Fruit peels, pulp, and seeds	Flavonol glycoside	Isorhamnetin‐3‐neohesperidoside	171
Fruit peels, pulp, and seeds	Flavonol pyrane	Quercetin 3‐*O*‐rhamnoside	172
Fruit peels, pulp, and seeds	Flavonol pyrane	Quercetin‐3‐glucoside	173
Fruit peels, pulp, and seeds	Flavonol pyrane	Astragalin	174
Fruit peels, pulp, and seeds	Flavonol pyrane	Hyperoside	175
Fruit peels, pulp, and seeds	Flavone pyrane	Orientin	176
Fruit peels, pulp, and seeds	Flavone glycoside	Isorhoifolin	177
Fruit peels, pulp, and seeds	Flavone glycoside	Linarin	178
Fruit peels, pulp, and seeds	Isoflavone pyrone	Puerarin	179
Peel skin	Methoxylated flavonol	5‐Hydroxy‐3,3′,4′,6,7‐pentamethoxy‐flavone	180
Peel skin	Methoxylated flavone	3′,4′,5,7,8‐Pentamethoxyflavone	181
Peel skin	Flavone	4′,5,7,8‐Tetramethoxyflavone	182
Peel skin	Flavone	3′,4′,5,6,7‐Pentamethoxyflavone	183
**Phenols**			
Fruit peels and pulp	Phenolic acid	Caffeic acid	184
Fruit peels and pulp	Phenolic acid	Ferulic acid	185
Fruit peels and pulp	Phenolic acid	Chlorogenic acid	186
Fruit peels and pulp	Cinnamic acid	*p*‐Coumaric acid	187
Fruit peels and pulp	Cinnamic acid	Sinapic acid	188
Fruit peels and pulp	Benzoic acid	Protocatechuic acid	189
Fruit peels and pulp	Phenolic acid	4‐Hydroxybenzoic acid	190
Fruit peels and pulp	Benzoic acid	Vanillic acid	191
Fruit peels, twig bark, and leaves	Phenolic aldehyde	4‐Hydroxybenzaldehyde	192
Fruit peels, twig bark, and leaves	Phenolic methanoate	Depcitrus B	193
Fruit peels, twig bark, and leaves	Depsides	Atranorin	194
Fruit peels, twig bark, and leaves	Depsides	Depcitrus A	195
Fruit peels, pulp, and seeds	Polyphenolic acid	Gallic acid	196
Fruit peels, pulp, and seeds	Benzoic acid	Syringic acid	197
Fruit peels, pulp, and seeds	Tannin	Valoneic acid dilactone	198
Fruit peels, pulp, and seeds	Phenolic acid	3‐*p*‐Coumaroylquinic acid	199
Fruit peels, pulp, and seeds	Benzoate	Methyl gallate	200
Fruit peels, pulp, and seeds	Polyphenol	Phloretin	201
Fruit peels, pulp, and seeds	Polyphenol glycoside	Phloridzin	202
Fruit peels, pulp, and seeds	Benzaldehyde	Syringaldehyde	203
Fruit peels	Phenolic acid	Methyl salicylate	204
**Alkaloids**			
Leaves	Pyrano[2,3‐a]acridone	Reticarcidone A	205
Leaves	Acridone alkaloids	5‐Hydroxynoracronycine	206
Leaves	Acridone alkaloids	Citracridone‐I	207
Leaves	Acridone alkaloids	Citracridone‐II	208
Leaves	Acridone alkaloids	Citracridone‐III	209
Leaves	Acridone alkaloids	5‐Hydroxyarboriarborine	210
Leaves	Acridone alkaloids	Citpressine‐I	211
Leaves	Acridone alkaloids	Glycofolinine	212
Leaves	Acridone alkaloids	Citrusamine	213
Leaves	Acridone alkaloids	1,6‐Dihydroxy‐2,3,4‐trimethoxy‐9(10*H*)‐acridone	214
Leaves	Acridone alkaloids	Caffeine	215
Fruit peels, twig bark, and leaves	Acridone alkaloids	Citramine	216
Fruit peels, twig bark, and leaves	Xanthine alkaloid	Citrusinine I	217
**Lipids**			
Stem bark	Esterol	Stigmasterol	218
Stem bark	Esterol	β‐Sitosterol	219
Stem bark	Fatty acid	Palmitic acid	220
Fruit peels	Fatty acid	Eicosanoic acid	221
Fruit peels	Esterol	β‐Sitosterol acetate	222
Fruit peels	Cholesterol	Cholest‐5‐en‐3‐ol	223
**Diverse classes**			
Fruit peels	Active form of vitamin E	α‐Tocopherol	224
Fruit peels, twig bark, and leaves	Oxygenated benzene	Sphaerophorol dimethyl ether	225
Fruit peel skin	Acyclic hydrocarbon	9‐Eicosyne	226
Fruit peel skin	Alcohol ester	1,2,3‐Propanetriol,1‐acetate	227
Fruit peel skin	Ester	Octadecanoic acid,2‐*oxo*‐methyl	228
Fruit peel skin	Carboxylic acid	9,12‐Octadecadienoic acid (*Z*,*Z*)	229
Fruit peel skin	Ester	9,12‐Octadecadienoic acid (*Z*, *Z*)‐, methyl éster	230
Fruit peel skin	Ester	Hexadecanoic acid, methyl ester	231
Fruit peel skin	Ester	[1,1′‐Bicyclopropyl]‐2‐octanoic acid, 2′‐hexyl‐, methyl ester	232
Fruit peel skin	Sulfhydryl	Methanecarbothiolic acid	233
Fruit peel skin	Carboxylic acid	Hexadecanoic acid	234
Fruit peel skin	Pyran	4*H*‐pyran‐4‐one, 2,3‐dihydro‐3,5‐dihydroxy‐6‐methyl	235
Fruit peel skin	Aldehyde	2‐Furancarboxaldehyde, 5‐(hydroxy)	236
Fruit peel skin	Alcohol	2‐Methoxy‐4‐vinylphenol	237
Fruit peel skin	Amide	Butyraldehyde, semicarbazone	238
Fruit peel skin	Olefin hydrocarbon	2,6‐Octadiene, 4,5‐dimethyl	239
Fruit peel skin	Dihydroxylated ester	Hexadecanoic acid, 2‐hydroxy‐1‐	240
Fruit peel skin	Ketone	Butylphosphonic acid, pentyl 4‐	241
Fruit peels	Aromatic hydrocarbon	Naphthalene	242
Fruit peels	Alcohol	Decanol	243
Fruit peels	Alcohol	Octanol	244


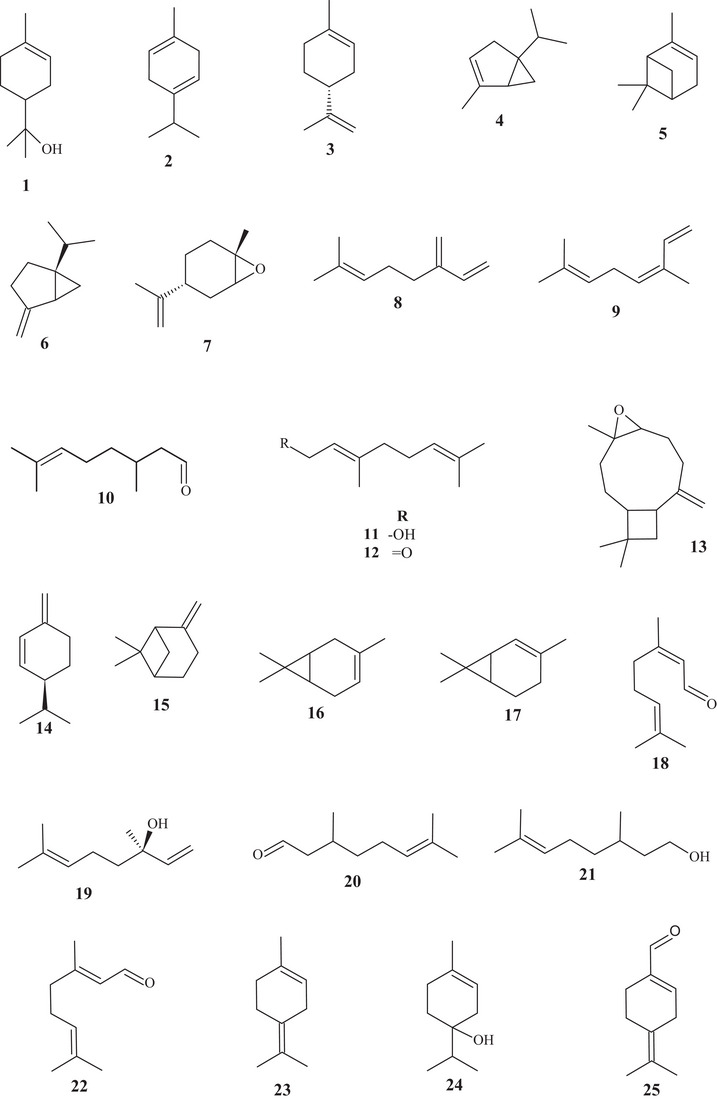



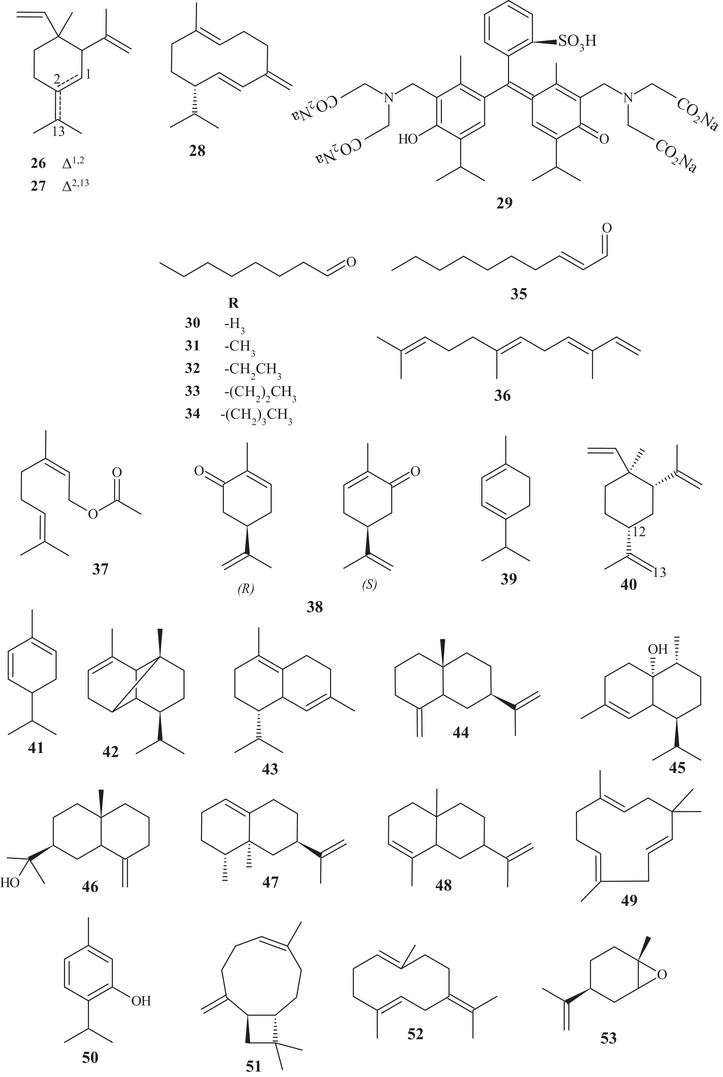



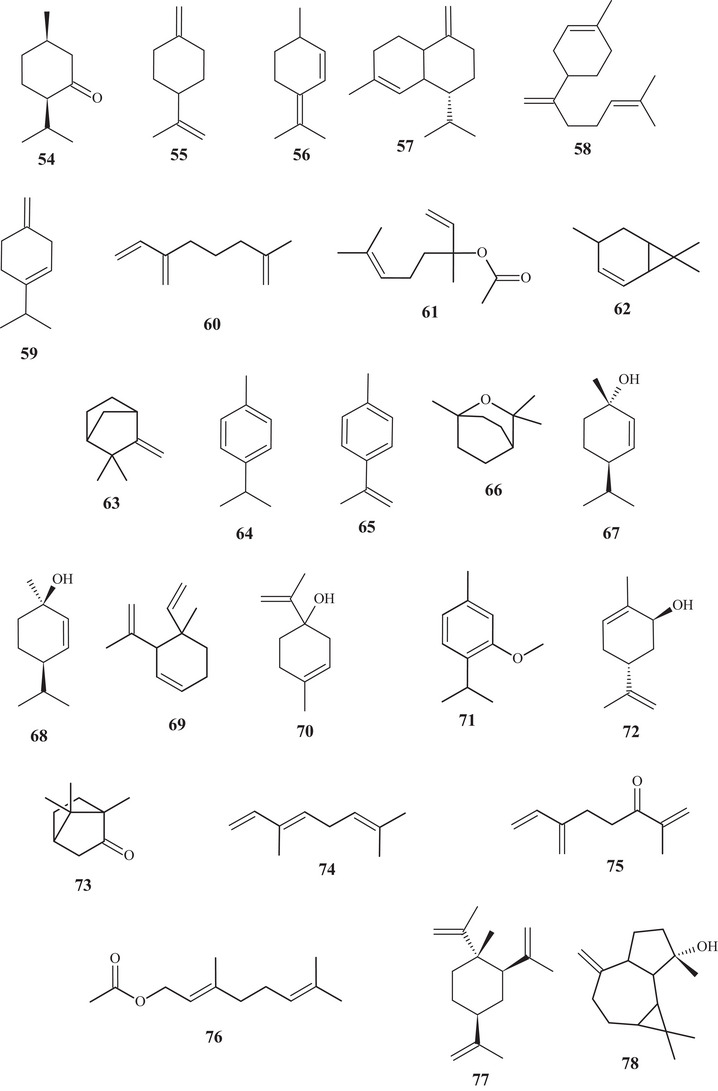



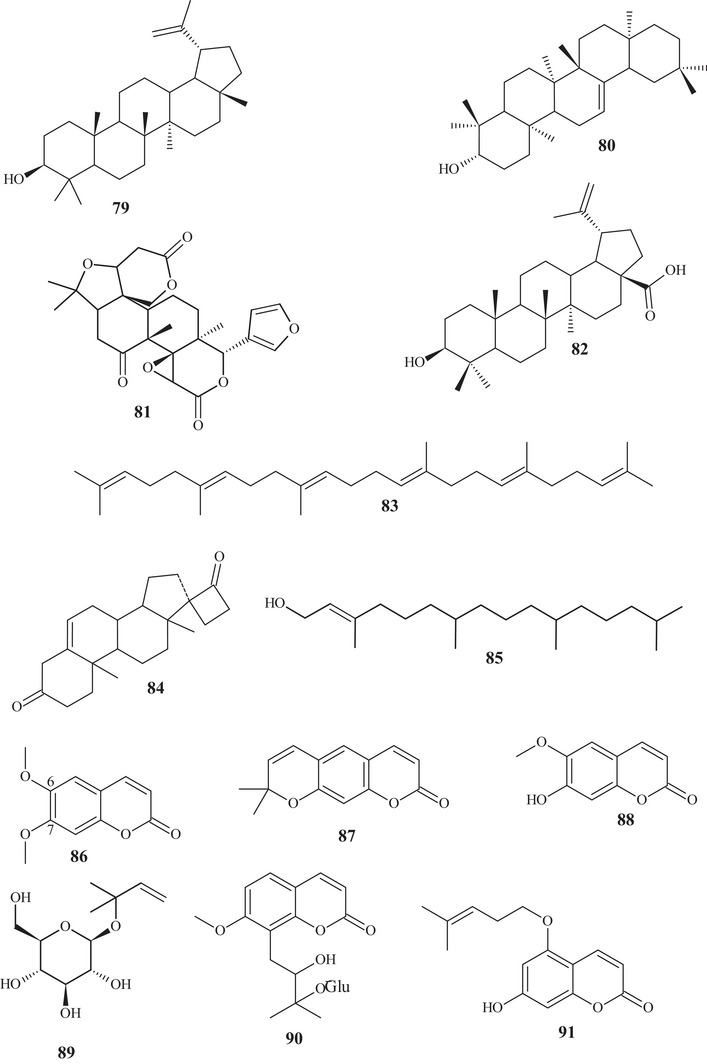



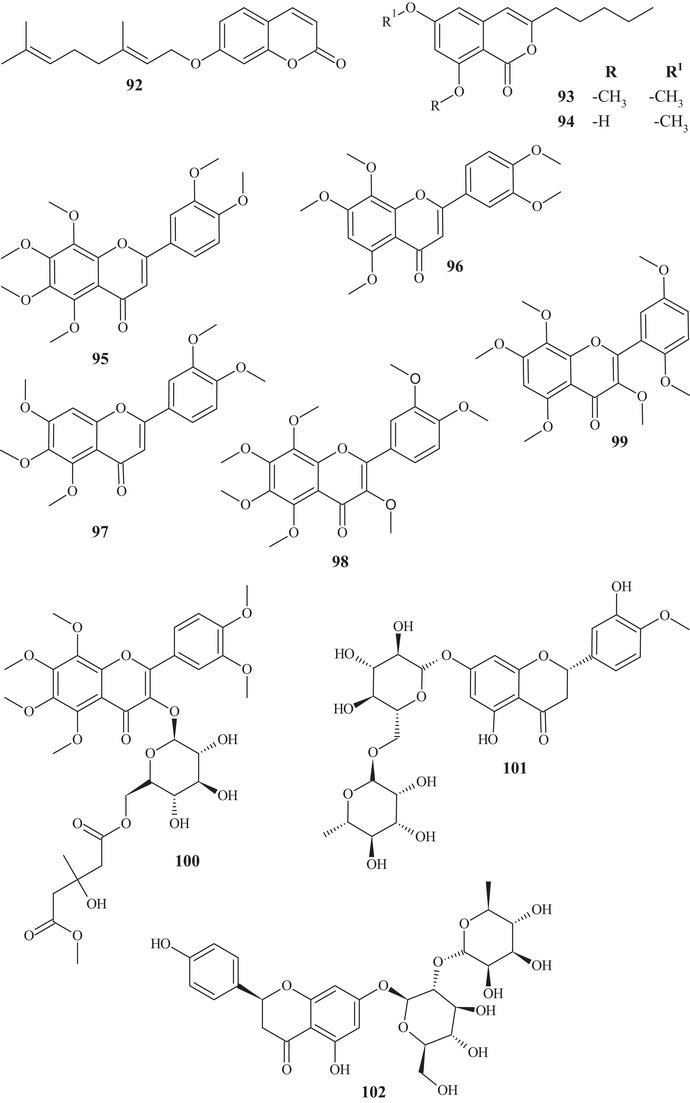



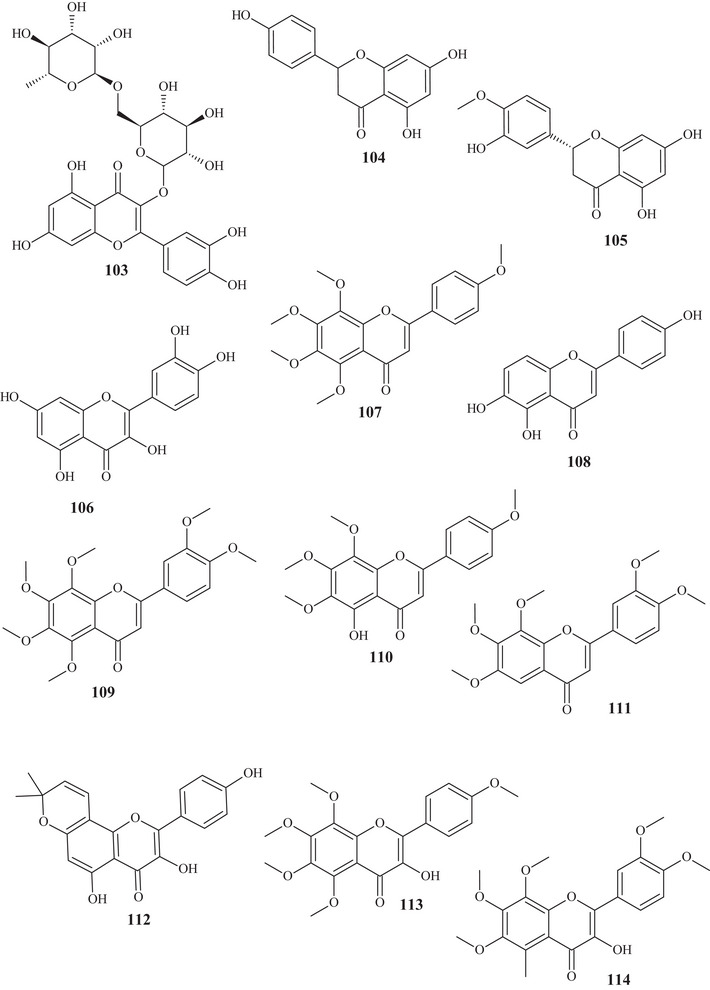



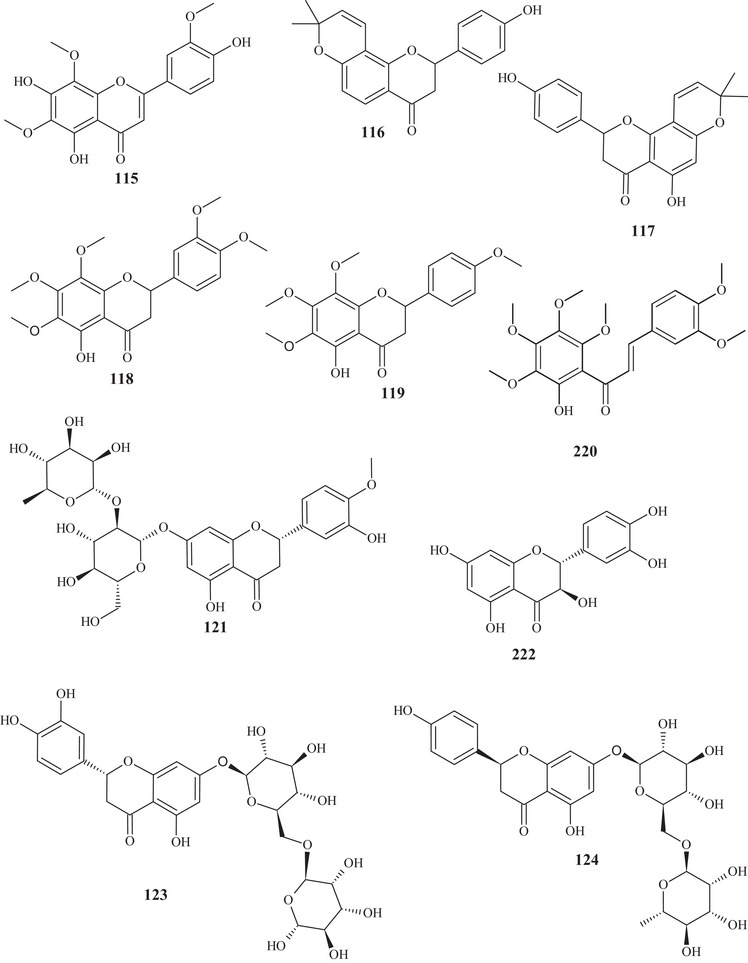



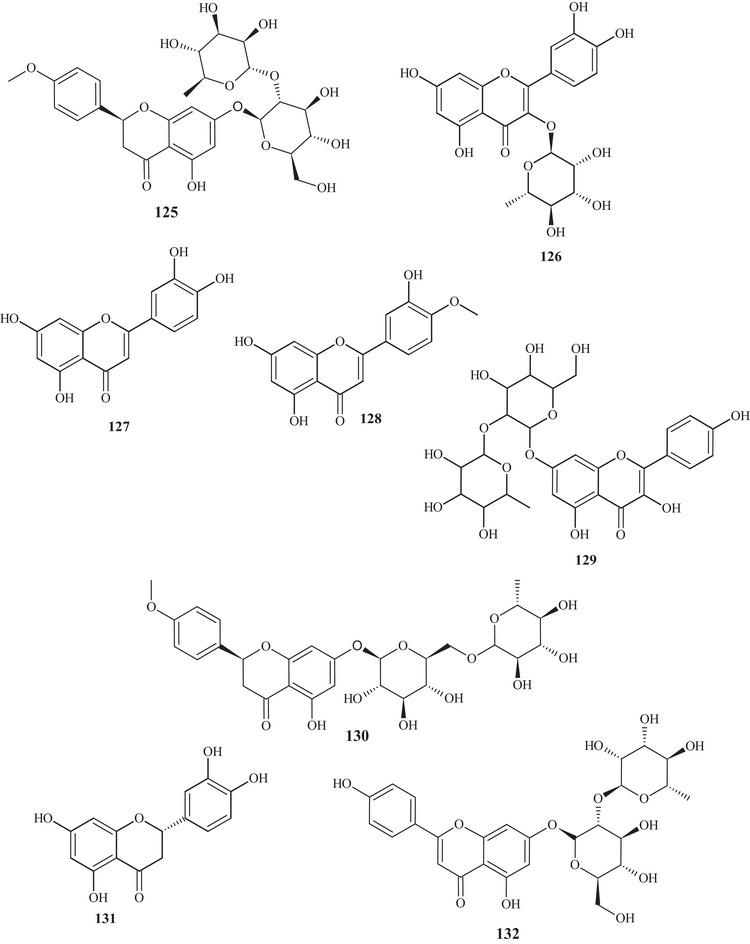



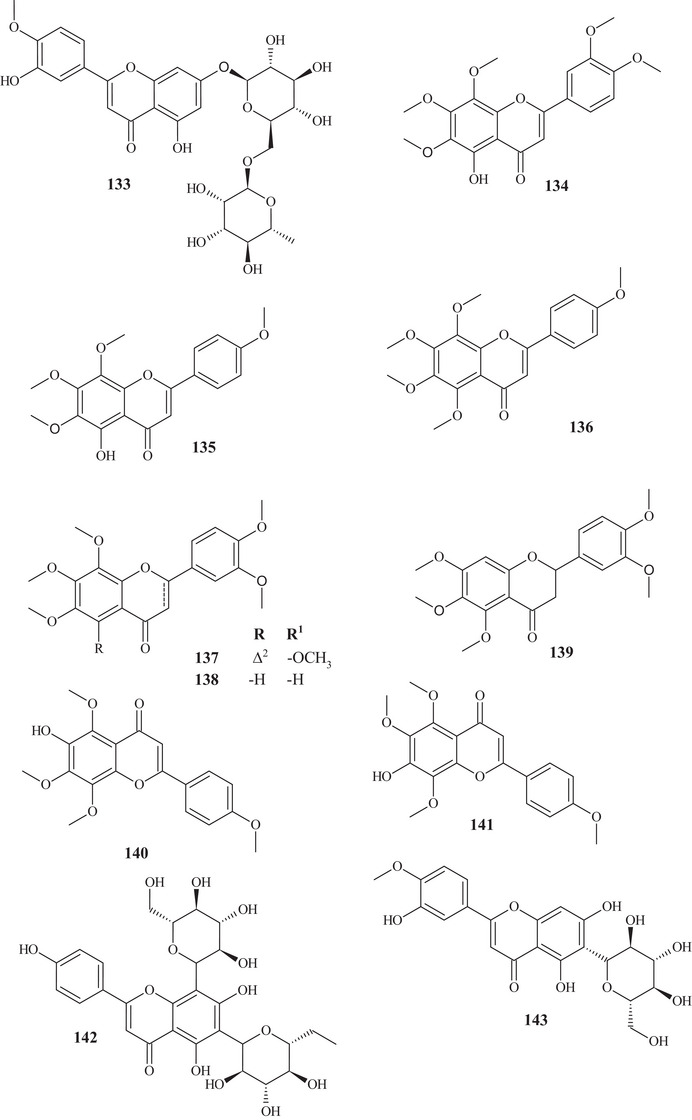



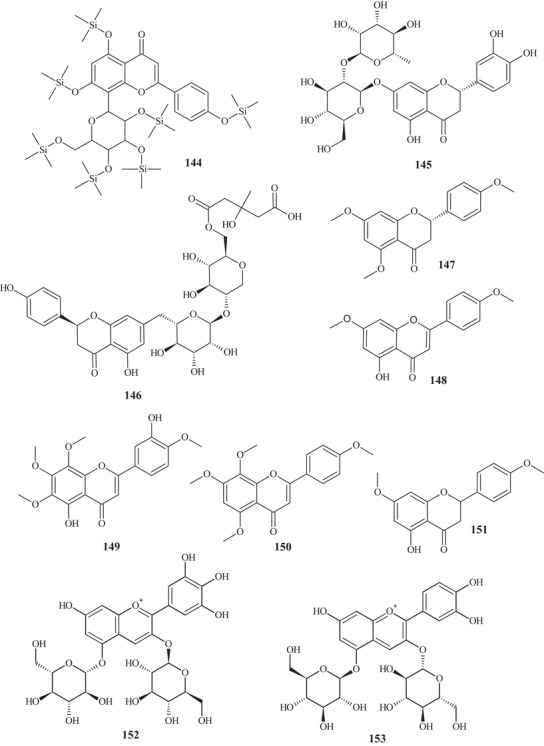



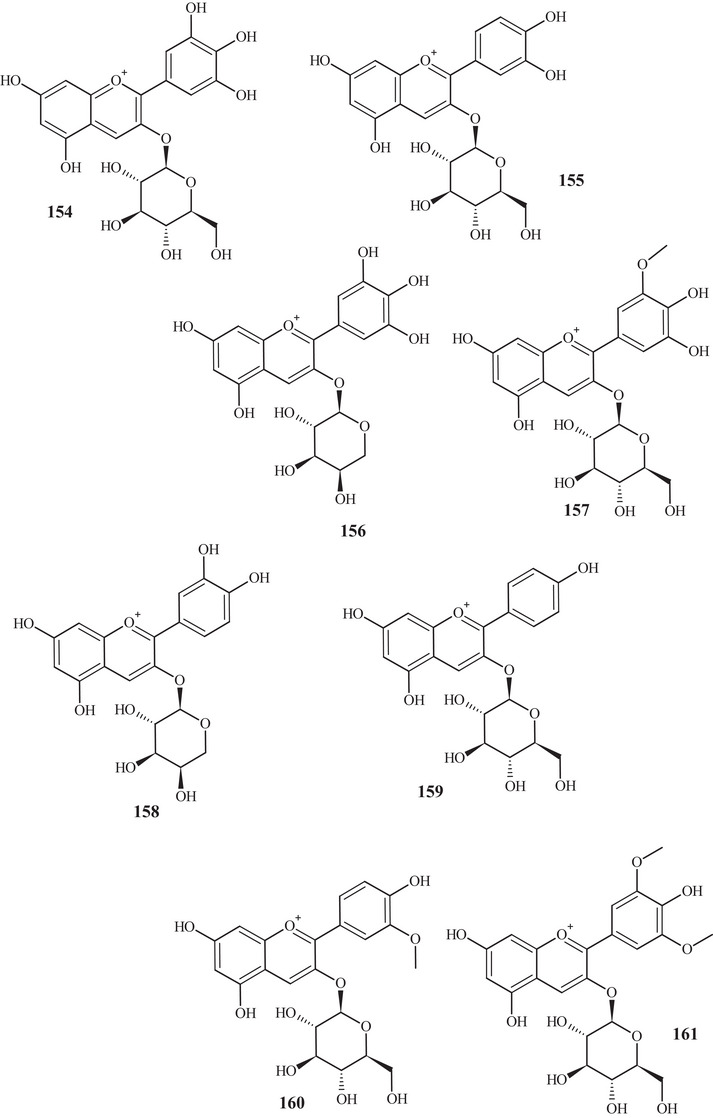



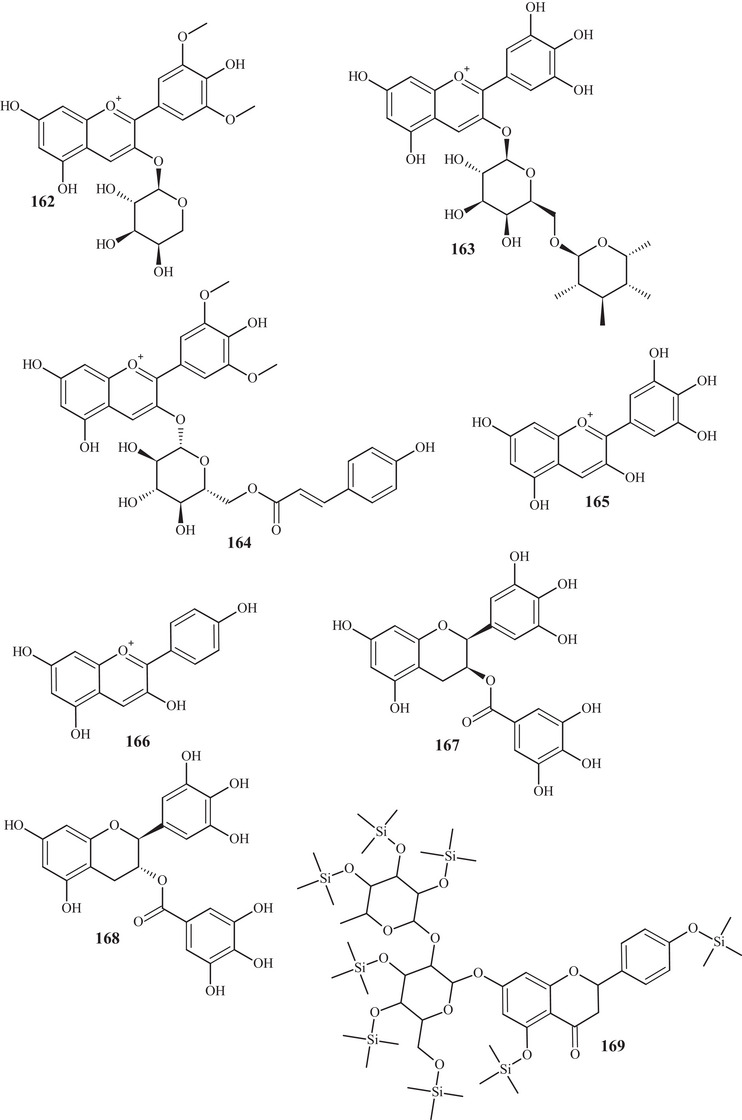



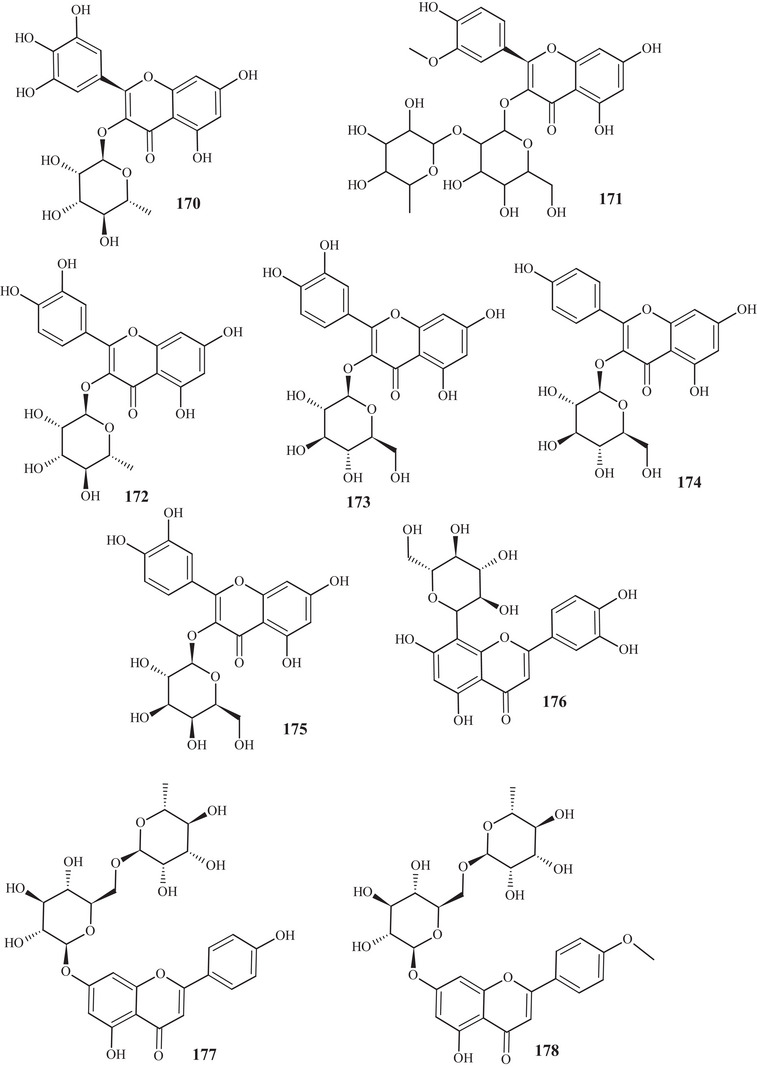



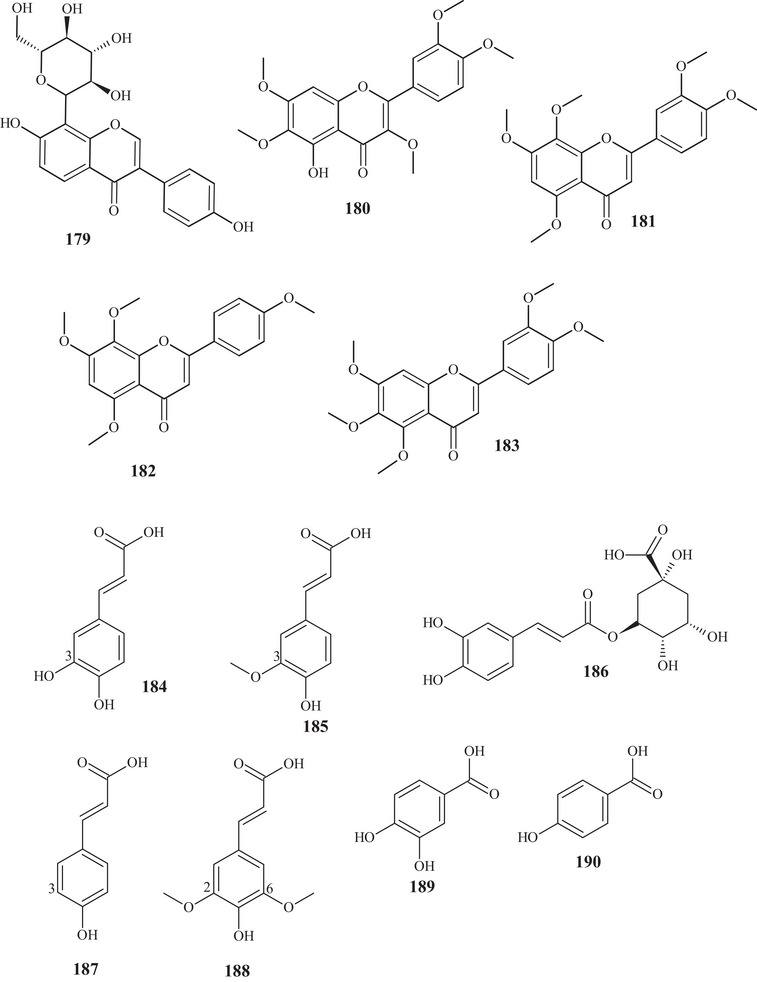



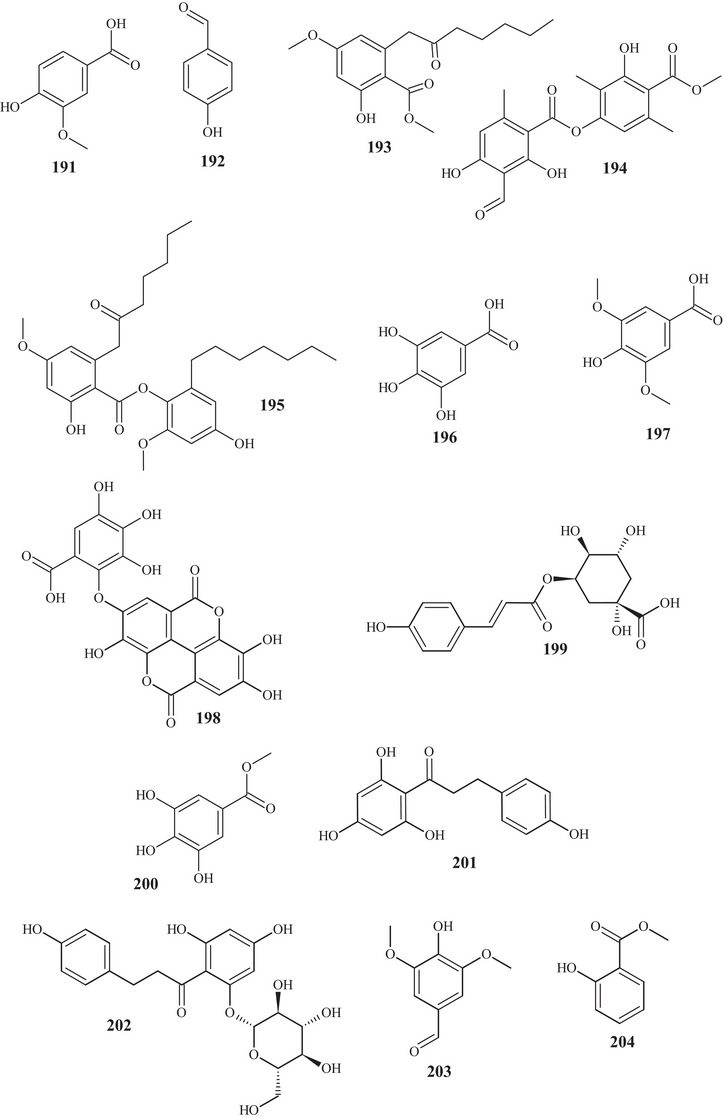



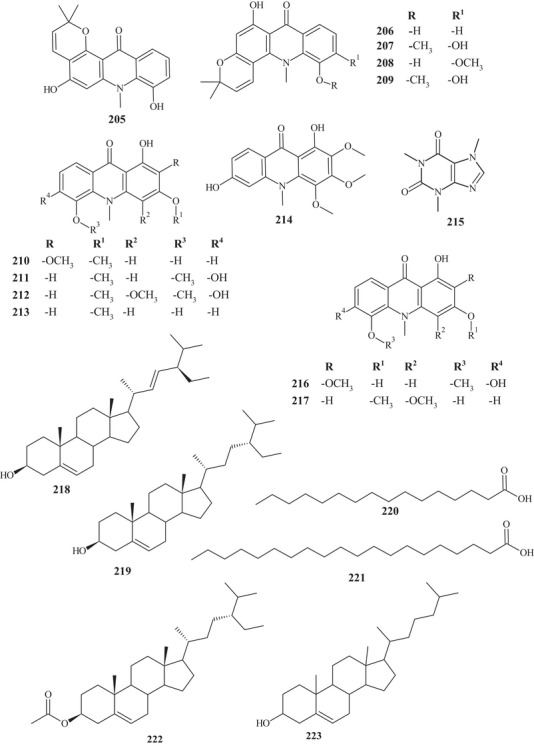



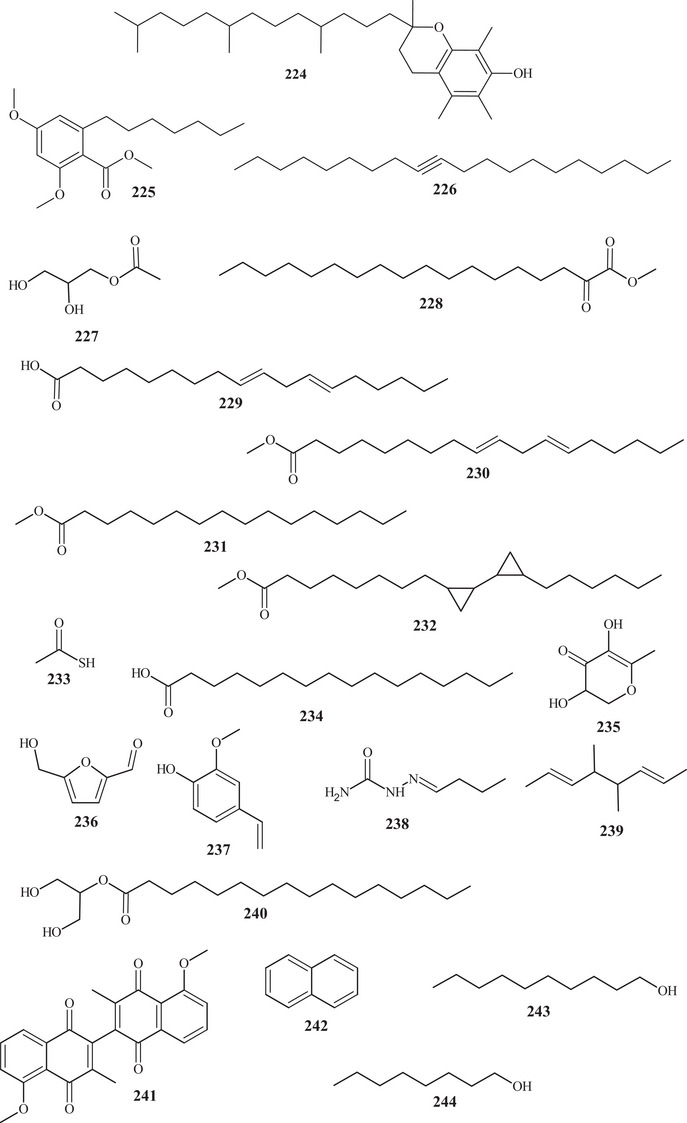


## 3 Biological Activities

### 3.1 Larvicidal Activity

Biopesticides derived from natural products have gained prominence as an innovative alternative for pest control in agriculture. Although they have been used as insecticides for more than 20 years, their full potential has not yet been fully described, but they are recognized for their safety and environmental sustainability. Monoterpenes, sesquiterpenes, and phenylpropanoids have broad application as bioactive agents and can be used either individually or incorporated into specific formulations. When applied directly to the target, these compounds act as pesticides, larvicides, enzymatic inhibitors, or repellents, interfering with essential physiological processes. Their mechanism of action involves disrupting cell membrane integrity, inhibiting metabolic enzymes, and altering hormonal pathways that compromise the development and growth of the target organism, resulting in paralysis, starvation, or rapid death. In addition, some of these compounds affect the nervous system and cellular respiration, reinforcing their effectiveness as biological pest control agents [40].

Oliveira et al. [14] in the chromatographic analysis identified 13 compounds (**1**–**13**) in the EO of *C. reticulata* Blanco fruit peels, totaling 97.8% of its composition. The main component was limonene (**3**) (85.7%), followed by γ‐terpinene (6.7%) (**2**) and myrcene (**8**) (2.1%). The larvicidal activity against *Aedes aegypti* exhibited a lethal concentration (LC_50_) of 58.35 µg mL^−1^ and reached 100% mortality at 150 µg mL^−1^, which can be justified by the compounds, mainly limonene (**3**) which can affect the physiological systems of the larvae, such as the inhibition of cell membrane function, interfering with permeability and causing cell dehydration.

The EOs of various *Citrus* species share chemical similarities, with monoterpenes (**3**, **5**, **14–16**) standing out, including limonene (**3**), as highlighted by Kwangjai et al. [16]. A high concentration of limonene was also observed in the fruit of *C. reticulata*, as reported by Tao and Zhou [17]. A more recent study (Prommaban and Chaiyana 2022) [41] involving microemulsions of EOs from *Citrus* peels and leaves also identified limonene (**3**) as a key compound responsible for biological activities. Castro et al. [42] describe that limonene (**3**) is partially responsible for larvicidal effects; its activity is mainly related to the disruption of cellular membrane integrity and larval cuticular structures, leading to ion loss and dehydration, which culminate in cell death.

### 3.2 Leishmanicidal Activity

Natural products, especially medicinal plant extracts, have shown promising leishmanicidal effects. The antiparasitic action in vitro and in vivo acts without causing toxicity to host cells. Furthermore, plants and their by‐products induce an increase in nitric oxide (NO) production in infected macrophages, enhancing the immunological response against the parasite. These findings highlight the potential of natural products in the development of new treatments for leishmaniasis, as pointed out by [43].

The studies by Oliveira et al. [14] demonstrated promising activity against promastigote forms of *Leishmania amazonensis*, with an IC_50_ value of 8.23 µg mL^−1^, a result considered highly active. However, the research notes that the EO from the peels of *C. reticulata* fruits may show variable results due to processing quality, as the extraction process often occurs without proper regulation. The assays indicate that the high antileishmanial activity observed in this study may be attributed to the significant presence of limonene (**3**), a monoterpene known for its action against *Leishmania parasites*. Limonene alters cellular membrane permeability, promoting redox imbalance in parasitic cells with increased reactive oxygen species (ROS), mitochondrial damage, and reduced ATP synthesis, ultimately leading to oxidative stress and cell death.

### 3.3 Anti‐Inflammatory Activity

Wen et al. [44] present a study suggesting that the inhalation of the EO from *C. reticulata* fruit peels has significant anti‐inflammatory effects on the airways and pulmonary emphysema in animal models of Chronic Obstructive Pulmonary Disease (COPD). The main results indicate a reduction in inflammation, possibly mediated by the modulation of macrophage activity, decreasing levels of IL‐18, IL‐17A, IL‐12p70, and GM‐CSF, suppressing the relative mRNA expression of interferon‐γ (IFN‐γ), IL‐4, and MMP‐12, and increasing IL‐10 mRNA expression, in addition to improving body weight and mental state of COPD rats; however, the study does not describe to which constituents this activity was attributed.

Hilel et al. [45] worked with the ethanolic extract of mature *C. reticulata* peels against the anti‐inflammatory effect on the mechanical hyperalgesia in a dextran sodium sulfate (DSS)‐induced colitis model; this showed systemic activity because nobiletin (**95**), a flavonoid found in the peels and in plants of this genus, has the ability to reduce several inflammatory markers in animal models of osteoarthritis. The study highlighted that naringenin (**104**) inhibited the elevation of inflammatory cytokine expression, which is aligned with the protective effect of the plant on intestinal barrier integrity.

Kwangjai et al. [16] evaluated the brain wave modifications and sleep parameters induced by the use of *C. reticulata* Blanco EO. The inhalation of the EO induced electroencephalogram patterns similar to the control, with significant changes in the delta, theta, alpha, beta1, and gamma frequency bands in the frontal and parietal cortex of rats, which confers anti‐inflammatory activity. Regarding sleep parameters, EO reduced REM sleep latency and increased both total sleep time and the number of sleep episodes, unlike diazepam, which increased wakefulness and non‐REM sleep episodes. This activity was attributed to *D*‐limonene (**3**) (95.7%) as the main component of *C. reticulata* EO, with α‐pinene (**5**) (0.5%), β‐pinene (**15**) (1.1%), and *δ*‐3‐carene (**16**) (1.5%) obtained by GC–MS analysis.

The dried pericarps of the species also demonstrated an inhibitory effect on foam cell formation derived from macrophages and promoted HDL‐mediated cholesterol efflux causing inflammation, exhibiting antiatherogenic capacity by interfering with RAW264.7 foam cell formation through inhibition of lipid uptake and promotion of HDL‐mediated cholesterol efflux, as described [46].

Duan et al. [34] investigated the polymethoxyflavones (PMFs) (**95**, **98**, **134**–**139**) present in the peels of *C. reticulata* fruits and isolated eight distinct PMFs, identified through nuclear magnetic resonance (NMR) and mass spectrometry techniques. Among these, the compound 6,7,8,3′,4′‐pentamethoxyflavanone (**138**) stands out, being isolated for the first time from the peels of this specific variety. The compound 3,5,6,7,8,3′,4′‐heptamethoxyflavone (**98**) demonstrated potent inhibitory activity against sterol regulatory element‐binding proteins, whereas 5‐hydroxy‐6,7,8,3′,4′‐pentamethoxyflavone (**134**) exhibited strong antiproliferative activity against tumor cell lines, with the ability to inhibit NO production. The efficacy varied mainly according to the number of methoxy groups present in the molecules, indicating anti‐inflammatory and antiproliferative properties.

Ferreira et al. [28] investigated the extraction efficiency of phenolic compounds from the peels of *C. reticulata* Blanco. The main compounds identified were hesperidin (**101**), rutin (**103**), naringenin (**104**), and tangeretin (**107**), which together accounted for approximately 86% of the total phenolics extracted from the species. In addition, the extracts demonstrated dose‐ and cell line–dependent antiproliferative activity, with IC_50_ values of 174.5 ± 5.8 µg mL^−1^ for BT‐474 cells (breast cancer), 391.9 ± 15 µg mL^−1^ for Caco‐2 cells (colorectal cancer), and above 500.00 µg mL^−1^ for HepG2 cells (liver cancer), indicating anti‐inflammatory activity. These compounds act by modulating the NF‐κB and MAPK pathways, reducing pro‐inflammatory cytokines and inducing apoptosis, which results in inhibition of cell growth, decreased tumor proliferation, and anti‐inflammatory action.

Hawas et al. [47] investigated flavonoids and their hepatoprotective efficacy against thioacetamide‐ (TAA‐) induced liver injury in rats. The isolated flavonoids showed a significant reduction in serum levels of hepatic enzymes (alanine aminotransferase [ALT], aspartate aminotransferase [AST]) and bilirubin, which had been elevated due to TAA administration. A decrease in tumor necrosis factor‐α (TNF‐α) levels and a significant reduction in hepatic hydroxyproline content were also observed, indicating reversal of induced fibrosis. Histological examination of liver tissues corroborated the anti‐inflammatory effect of the ethanolic extract, showing improved cellular integrity and reduced tissue damage.

Wu et al. [48] evaluated the protective effect of the flavonoid composition from the peels of *C. reticulata*. The levels of pro‐inflammatory cytokines, including TNF‐α, IFN‐γ, interleukin‐1β (IL‐1β), and interleukin‐6 (IL‐6), were significantly reduced in mice treated with the flavonoid solution. Regarding the hepatoprotective effect, there was a significant reduction in serum levels of ALT and AST, enzymes indicative of liver injury. The peel extract contains flavonoids such as narirutin (**124**) (10 mg g^−1^), hesperidin (**101**) (84.9 mg g^−1^), nobiletin (**95**) (27.3 mg g^−1^), tangeretin (**107**) (28.3 mg g^−1^), and 5‐demethylnobiletin (**111**) (8.4 mg g^−1^), which are responsible for the protective activity.

The ethanolic extract of *C. reticulata* pericarp also showed anti‐inflammatory activity. The expressions of type I and III collagen proteins were suppressed, suggesting an effective inhibition of collagen deposition in lung tissue, resulting in the suppression of pyridinoline production, a collagen cross‐linking marker, in addition to a decrease in the protein expressions of transforming growth factor beta 1 (TGF‐β1) and Smad3 in the lungs. These findings suggest antifibrotic properties, acting in the inhibition of collagen synthesis, cross‐linking, and deposition, possibly mediated by the downregulation of the TGF‐β1/Smad3 pathway, as pointed out by Li et al. [49].

The scientific evidence reported by Srimathi and Gurunathan [26] on the methanolic extracts from the fruit peels and seeds of *C. reticulata* describes their ability to neutralize toxins from the Indian cobra *Naja naja* venom. The peel extracts of *citrus* species significantly inhibited the phospholipase A_2_ (PLA_2_) (75%), protease (71%), and hemolytic (80%) activities induced by the venom. The presence of β‐sitosterol (**219**), hexadecanoic acid (**240**), eicosanoic acid (**221**), α‐tocopherol (**224**), and squalene (**83**) contributes to the neutralization of the venom's cytotoxic and inflammatory effects.

The effects of the ethanolic extract from the dried peels of *C. reticulata* on the inflammatory response of macrophages, as reported by Lee et al. [50], showed a significant decrease in the production of the pro‐inflammatory cytokine IL‐6, while stimulating a low concentration of lipopolysaccharide (LPS) (1 ng mL^−1^) and increasing the expression of Ras guanine nucleotide–releasing protein 3 (RasGRP3). Nobiletin (**95**) was described in the study as one of the main components contributing to the observed anti‐inflammatory effects, modulating the production of pro‐inflammatory cytokines in macrophages.

Zhou et al. [51] isolated and purified a predominant polysaccharide from the peels of *C. reticulata*, identified as an arabinogalacturonan. The structure of the polysaccharide was determined by NMR spectroscopy, revealing a main chain of (1 → 4)‐α‐d‐galacturonan with α‐l‐arabinofuranosyl side branches. This compound showed no toxicity toward macrophages; however, it significantly promoted NO production in a dose‐dependent manner, indicating potential immunomodulatory activity.

### 3.4 Antioxidant Activity

Ferreira et al. [28] demonstrated that there are no significant differences in the extraction efficiency of phenolic compounds when two different solvents are used. The main compounds identified in the fruit peel extracts, ethanolic and aqueous, were hesperidin (**101**), naringenin (**104**), tangeretin (**107**), and rutin (**103**), which together represented approximately 86% of the total extracted phenolics. Furthermore, the application of solid‐phase extraction allowed a 4.5‐fold enrichment in phenolic compounds, and the antioxidant activity of the extracts presented an IC_50_ of 174.5 ± 5.8 µg mL^−1^ (BT‐474), 391.9 ± 15 µg mL^−1^ (Caco‐2), and >500.00 µg mL^−1^ (HepG2).

*Citrus* compounds may have different chemical contents when found in different tissues. Zhang et al. [52] highlight that fruit peels showed the highest average total phenolic contents (27.18 mg gallic acid equivalents per gram dry weight) and total flavonoids (38.97 mg rutin equivalents per gram dry weight), whereas pulp, seed, and juice residues: The contents varied among the different genotypes analyzed, with values lower than those found in the peels. As for antioxidant capacity, the peels showed the highest antioxidant capacity, with DPPH^•^ values equivalent to 21.92 mg of ascorbic acid equivalent antioxidant capacity per gram dry weight and ABTS^•+^ values equivalent to 78.70 mg per gram dry weight. In other tissues, the antioxidant capacity varied, generally being lower than that observed in the peels.

The findings of Zhou et al. [51] described the structural variations of polysaccharides present in the peels of *C. reticulata*. A linear (1 → 4)‐α‐d‐galacturonan structure was isolated and characterized, revealing significant immunomodulatory activity in two distinct polysaccharides derived from this backbone—one of the arabinogalactan type and the other of the arabinogalacturonan type. These compounds exhibited antioxidant and immunoregulatory properties, demonstrating their ability to protect cells against hydrogen peroxide–induced oxidative damage by enhancing the activity of antioxidant enzymes.

The investigations by Mahdi et al. [53] suggest improvements in the antioxidant properties of nanocapsules containing *C. reticulata* EO (CEO) through the addition of *Cinnamomum verum* (cinnamon) and *Dianthus caryophyllus* (clove) EOs. The incorporation of cinnamon and clove EOs increased the total phenolic content of the nanocapsules by 1.75 and 1.54 times, respectively, compared to nanocapsules containing only CEO. The addition of cinnamon and clove oils resulted in a significant increase in the antioxidant activity of the nanocapsules, increasing it by 3.57 and 2.08 times, respectively.

Zang et al. [54] investigated the phenolic compounds present in the methanolic extract from the fruit peels of 14 wild Citrus genotypes. Among the main phenolic compounds identified were nobiletin (**95**), hesperidin (**101**), eriocitrin (**123**), narirutin (**124**), and ferulic acid (**185**), which were responsible for the combined antioxidant potency index (APC), showing significant variations ranging from 58.84 to 98.89 among the analyzed species. The mechanism of action of these compounds involves electron or hydrogen atom donation to neutralize ROS, as well as metal ion chelation that catalyzes oxidative reactions. They also modulate endogenous antioxidant enzymes (such as SOD, CAT, and GPx), resulting in reduced oxidative stress and protection of cellular structures against oxidative damage.

Prommaban and Chaiyana [41] presented limonene (**3**) as the main component, varying between 72.5% and 95.7%, depending on the source (fresh leaves, fresh peels, or dried peels). The oils from the leaves and fruit peels of *Citrus* species (*C. aurantifolia* and *C. reticulata*) demonstrated strong antioxidant activities, including the ability to neutralize free radicals and inhibit lipid peroxidation. The oil from dried *C. reticulata* peels significantly inhibited collagenase and tyrosinase activities, suggesting potential for reducing wrinkles and skin blemishes, significantly reducing skin irritation in human volunteers, indicating that microemulsification can improve the safety of topical use of these EOs.

Najem et al. [55] elucidated that the EO from the fruit peels exhibited higher antioxidant activity when compared to the pure major compound. The chemical profile analyzed by GC/MS identified limonene (**3**) as the main component (81.90%). The EO demonstrated a potent antioxidant effect in the DPPH^•^, ABTS^•+^, and FRAP assays, with mean inhibitory concentrations (IC_50_) of 2.01, 3.47, and 4.71 mg mL^−1^, respectively. The mechanism of action involves electron and hydrogen atom donation to neutralize free radicals (such as DPPH^•^ and ABTS^•+^), in addition to the reduction of ferric ions (Fe^3+^ → Fe^2+^) in the FRAP assay. This combined action promotes the stabilization of reactive radicals, interruption of oxidative chain reactions, and protection of biomolecules against oxidative stress, thereby justifying the high antioxidant capacity observed.

The polysaccharides from *C. reticulata* fruit peels demonstrated significant antioxidant activities, including the ability to reduce ferric ions (FRAP), neutralize hydroxyl radicals (^•^OH), DPPH^•^ radicals, and superoxide radicals (O_2_^−^), in a concentration‐dependent manner. These activities suggest that polysaccharides can act as effective natural antioxidants [56].

The peel oil of *C. reticulata* may act as an antiatherogenic agent by modulating oxidative stress. Molecular docking analyses suggested that the oil's compounds—such as α‐farnesene (**36**) and neryl acetate (**37**), as well as the major component limonene (**3**) and its metabolites—may inhibit lanosterol synthase, contributing to reduced cholesterol synthesis and decreasing the formation of foam cells, which are precursors of atherosclerotic plaques. In addition, the oil prevented copper sulfate–induced LDL lipid peroxidation, as described by Castro et al. [42].

Xi et al. [32] investigated the phenolic composition and antioxidant properties of the fruit pulps from 14 *C. reticulata* Blanco genotypes. The chemical profile identified hesperidin (**101**) as the predominant flavonoid, whereas ferulic acid (**185**) was the most abundant phenolic acid in the methanolic extract of *C. reticulata* peels. Overall, the pulps of the Nieduyeju, Guangxihongpisuanju, and Wangcangzhoupigan genotypes contain higher levels of phenolic compounds and exhibit greater antioxidant capacities compared with Satsuma and Ponkan, indicating their potential as rich sources of phytochemicals and natural antioxidants. These effects reduce oxidative stress, protect lipids, proteins, and DNA from damage, and maintain cellular redox balance, supporting the high antioxidant capacity observed in pulps richer in phenolics.

Job et al. [20] found that the antioxidant properties of the EO from fruit peels were attributed to the presence of compounds such as *D*‐limonene (**3**), α‐pinene (**5**), α‐myrcene (**60**), and *cis*‐terpinene (**39**) which have radical scavenging capacity, ferric reducing antioxidant power, and lipid peroxidation inhibition, demonstrating that the thick outer layer that is often discarded due to its low economic value generally contributes to potential health benefits.

Rashed et al. [57] in GC–MS analysis revealed *D*‐limonene (**3**) as the main component of *C. reticulata* leaf EO, with concentrations ranging between 50.3% and 65.7%, depending on the applied treatment. Nanoemulsions developed using octenyl succinic anhydride modified starch (OSA‐MS) and almond oil demonstrated significant antioxidant activity in the β‐carotene/linoleic acid system, with low degradation rates, indicating high resistance to oxidation compared to the control. These findings suggest that the combination of enzymatic pretreatment and ultrasound‐microwave techniques improves the extraction efficiency of *C. reticulata* leaf EO.

There is a relationship between the impact of different drying techniques on flavonoid, total phenol contents (TPCs), and the antioxidant capacity of green *C. reticulata* Blanco fruits. In the findings of Kumar et al. [33], hesperidin (**101**) was the predominantly identified flavonoid; lyophilized fruit samples with 12 and 14 mm diameter showed the highest contents, with 27.03% and 27.20%, respectively. In contrast, the same samples subjected to oven drying showed a reduced content of 17.99%. Lyophilized fruits exhibited a TPC: ranging between 50.50 and 54.19 mg of gallic acid equivalents per liter. It was also possible to observe a significant positive correlation between hesperidin (**101**) contents and antioxidant assay results.

Costanzo et al. [36] demonstrated that antioxidant capacity was higher in green fruits, whereas seeds significantly increased their antioxidant activity during maturation, reaching their peak in fully ripe fruits. The fruit peels exhibited the highest levels of polyphenols and flavonoids, whereas the seeds contained ascorbic acid and condensed tannins. These antioxidant effects are attributed to compounds such as anthocyanins, naringin (**102**), rutin (**103**), tannins, polyphenols, delphinidin‐3‐*O*‐glucoside (**154**), cyanidin‐3‐*O*‐glucoside (**155**), quercetin‐3‐glucoside (**173**), and gallic acid (**196**), which are responsible for chelating pro‐oxidant metals.

Phenolic compounds are described as the main contributors to the antioxidant capacity of citrus fruits due to their actions in neutralizing free radicals, donating electrons or hydrogen atoms, and chelating pro‐oxidant metals. It is worth highlighting the flavanones hesperidin (**101**), naringin (**102**), neohesperidin (**121**), didymin (**130**), and poncirin (**132**), which are present in *C. reticulata*. The study by Wang et al. [1] identified 39 flavonoid compounds, including 4 flavones, 9 flavanones, and 26 PMFs.

Apraj and Pandita [38] investigated the anti‐aging potential of *C. reticulata* Blanco fruit peels through alcoholic extracts obtained by two methods: Soxhlet and maceration. The extract obtained by Soxhlet showed phenolic compounds and flavonoids when compared to maceration. The extracts demonstrated significant antioxidant capacity in various assays due to the presence of PMFs. The alcoholic extract showed an EC_50_ value of 250.33 ± 40.16 µg mL^−1^, indicating strong DPPH^•^ free radical scavenging activity, an EC_50_ value of 221.27 ± 11.25 µg mL^−1^, suggesting effectiveness in neutralizing superoxide anions, in addition to inhibiting enzymes such as collagenases and elastase. Both alcoholic extracts of fruit peels showed similar activities in the ABTS^•+^ assay.

Phetkul et al. [25] report the isolation of a new depside, named depcitrus A (**195**), along with 31 already known compounds, from *C. reticulata* Blanco fruit peels, leaves, and branch barks. In this study, they found eight polymethoxy flavonoids, six acridone alkaloids, four flavonoids, four flavonoid glycosides, four coumarins, two isocoumarins, two depsides, two resorcinol derivatives, one coumarinic glycoside, one limonoid, one triterpenoid, and three benzoic derivatives. These compounds were evaluated for their ability to neutralize the DPPH^•^ free radical, and the results showed that the activity was very weak, with DPPH^•^ scavenging percentages ranging between only 0.65% and 23.2%.

Some studies evaluate antioxidant activity modulated by abiotic factors. Costanzo et al. [37] investigated how light‐emitting diode (LED) radiation applied after harvest can affect and modulate the levels of antioxidant compounds in *C. reticulata* fruits. LED light significantly modulated the levels of antioxidant compounds such as phenolics and carotenoids, leading to an increase in the total antioxidant activity of the fruits (peels), evidenced by free radical scavenging tests. These effects indicate that LED light can strengthen the natural defense mechanisms of the fruits and prolong their quality and shelf life during storage.

Lin et al. [21] investigated the chemical composition and antioxidant activity of EOs extracted from the fruit peels of four varieties of species within the *Citrus* genus. The chemical profile revealed that monoterpene hydrocarbons were the main components in all EOs, with limonene (**3**) being the predominant compound. All EOs demonstrated dose‐dependent DPPH^•^ and ABTS^•+^ free‐radical scavenging capacity, emphasizing that *Citrus* species may have potential applications in the pharmaceutical and food industries by stabilizing formed free radicals and protecting biomolecules.

Bhandari et al. [22] also described the antioxidant activity, which was superior to that of the positive control, quercetin (5.60 µL mL^−1^). *C. reticulata* showed the second‐best activity in the DPPH^•^ radical scavenging assay. This activity can be justified by GC–MS analysis which revealed limonene (**3**) as the predominant compound in all EOs, ranging from 63.7% to 89.1%. Other major constituents included γ‐terpinene (**2**) (0.24%–6.43%), β‐pinene (**15**) (0.15%–6.09%), and linalool (**19**) (0.35%–3.5%).

### 3.5 Antimicrobial Activity

Oliveira et al. [14] described that the EO from the fruit peels of *C. reticulata* exhibited promising activity against promastigote forms of *L. amazonensis*, with a 50% inhibitory concentration (IC_50_) of 8.23 µg mL^−1^. This activity is justified by its composition of 85.7% limonene (**3**), 6.7% γ‐terpinene (**2**), and 2.1% myrcene (**8**), which have the ability to alter plasma membrane permeability and fluidity, leading to the loss of essential cellular components and collapse of the microorganism's ionic homeostasis.

The literature describes findings of microorganisms acting in the modulation of abiotic stress on the development of *Citrus* species. Hasan et al. [58] evaluated partial tolerance to abiotic stresses in *C. reticulata* through *Agrobacterium*‐mediated transformation. Transgenic seedlings showed greater tolerance to drought and salinity compared to control plants, indicating that the microbial genes PsCBL and PsCIPK contributed to resistance to these abiotic stresses. These results point to genetic modification as a promising strategy to develop varieties more adapted to adverse environmental conditions.

The antifungal activity of the EO from *C. reticulata* fruit against *Penicillium* sp. was described by Tao and Zhou [17]. The findings report the dose‐dependent inhibition of *Penicillium italicum* and *Penicillium digitatum* growth. The antifungal activity was attributed to compounds such as limonene (**3**), citral (**18** and **22**), linalool (**19**), and terpenes. The fruit oil also caused structural damage to hyphae, increased membrane permeability, and loss of cellular components, suggesting cytotoxic action by compromising the integrity of fungal cells.

Wu et al. [30] also evaluated the antifungal activity of polymethoxylated flavones extracted from *C. reticulata* fruit peels. The study identified tangeretin (**107**) and nobiletin (**95**) as the main polymethoxylated flavones. The ethanolic extract showed high efficacy against *Aspergillus niger*, with a minimum inhibitory concentration (MIC) of 0.12 mg mL^−1^, significantly lower than that of isolated tangeretin (**107**) (1.5 mg mL^−1^). The data indicate changes in the fungal cell membrane, increasing potassium loss and electrical conductivity, reducing chitin production, and weakening the cell wall.

The EOs from the fruit peels of *Citrus aurantiifolia* and *C. reticulata*, which contain a high content of limonene (**3**), exhibited antimicrobial activity. Among the limonene enantiomers, (*R*)‐limonene showed greater antimicrobial action in internal exposure assays. The data indicate that these oils act through multiple mechanisms, such as disruption of the cell membrane and volatile action, and they have potential for use as antimicrobial agents, as noted by Fouad and Camara [19].

The EO obtained from the fruit peels of *C. reticulata* exhibited antibacterial activity against microorganisms such as *Escherichia coli*, *Pseudomonas aeruginosa*, *Staphylococcus aureus*, and *Salmonella enterica*, as demonstrated through agar diffusion assays and determination of the MIC. These findings indicate that the EO from *C. reticulata* peels has promising therapeutic potential, as it acts by disrupting the bacterial cell membrane, causing leakage of ions and cytoplasmic contents, in addition to enzymatic inhibition of energy metabolism. These effects are attributed to its main constituents: *S*‐limonene (**3**), α‐pinene (**5**), *cis*‐terpinene (**39**), and α‐myrcene (**60**), according to Job et al. [20].

The ethanolic extract obtained from the fruit peels of *C. reticulata* Blanco, rich in polymethoxylated flavones such as nobiletin (**95**) and tangeretin (**107**), contains compounds known for their antimicrobial activity by inhibiting enzymes involved in nucleic acid and protein synthesis, interfering with biofilm formation, and suppressing bacterial multiplication. Genotoxicity assays revealed that the extract did not induce mutations in bacteria nor cause genetic alterations in animal models, which reinforces its potential as a safe therapeutic agent, including for antimicrobial applications, as reported by Nakajima et al. [35].

The study by Phetkul et al. [25] identified a new compound from the depside class, named depcitrus A (**195**), isolated from different parts of the plant *C. reticulata* Blanco, in addition to several other previously known metabolites. Among the isolated compounds, some were subjected to antimicrobial activity tests, demonstrating the ability to inhibit the growth of pathogenic microorganisms such as *S. aureus* by disrupting the integrity of the bacterial cell wall and membrane, leading to membrane depolarization. These findings reinforce the potential of the phenolic compounds present in *C. reticulata* as natural agents with antimicrobial activity.

The EOs from the fruit peels of four species (*C. reticulata* Blanco cv, *C. reticulata* Blanco, *Citrus japonica* Thunb, and *C. sinensis* Osbeck cv. Newhall) were tested against five bacteria: *Bacillus subtilis*, *S. aureus*, *E. coli*, *P. aeruginosa*, and *Salmonella typhimurium*. The EOs exhibited varying antibacterial activities, with MIC values differing for each species. Among these species is *C. reticulata*, whose EO destabilizes the bacterial cell membrane, increases its permeability, and causes leakage of ions and proteins, ultimately leading to metabolic inhibition and cell death, as reported by Lin et al. [21].

Bhandari et al. [22] also evaluated the antibacterial activity of four species: *C. grandis* Osbeck red pulp, *C. reticulata* Blanco, *C. sinensis* Osbeck, and *C. grandis* Osbeck white pulp. The EOs were tested against the bacteria *S. aureus*, *E. coli*, *Klebsiella pneumoniae*, *Shigella sonnei*, and *Salmonella typhi* using the disk diffusion method. The results indicated that the oils exhibited antibacterial activity, justified by their interaction with bacterial membrane lipids, resulting in the loss of essential intracellular components. The EO of *C. reticulata* Blanco showed notable activity against *S. typhi*, reducing the MIC from 19 to 10 mm compared with the neomycin control.

The antibacterial activity and synergistic interaction of citrus EOs and limonene (**3**) with gentamicin against clinical isolates of methicillin‐resistant *S. aureus* (MRSA) were also evaluated. Sreepian et al. [15] identified 12 compounds in the EO extracted from the fruit peels of *C. reticulata*, with limonene (**3**) being the predominant component responsible for destabilizing the lipid bilayer of the bacterial membrane. Antibacterial assays revealed that both the oil and limonene (**3**) exhibited significant inhibitory effects against clinical isolates of MRSA and MSSA, with MICs ranging from 1.0 to 32.0 mg mL^−1^. The combination of gentamicin with the oil or with limonene (**3**) resulted in a synergistic interaction against MRSA and MSSA isolates (FIC indices: 0.012–0.258 and 0.012–0.375).

Mahdi et al. [53] investigated the enhancement of EO nanocapsules from *C. reticulata* fruits (CEO) through the addition of EOs from *C. verum* (cinnamon) and *D. caryophyllus* (clove), resulting in a significant increase in antifungal activity against different species. Against *Candida albicans*, antifungal efficacy increased 3.13‐fold with *C. verum* oil and 2.43‐fold with *D. caryophyllus* oil; for *Zygosaccharomyces rouxii*, a 1.24‐fold increase was observed with both oils. In the case of *A. niger*, activity increased 2.76‐fold with *C. verum* oil and 1.32‐fold with clove oil, whereas for *Penicillium roqueforti*, the increase reached 2.97‐fold with *cinnamon* oil and 2.09‐fold with clove oil. These results indicate a potential synergy between the EOs, promoting greater fungal membrane disruption due to increased cellular permeability and enzymatic protein denaturation, leading to greater inhibition of pathogenic fungal growth and making the nanocapsules more effective for fungal control.

### 3.6 Other Findings

Ye et al. [29] explored the chemical composition of the methanolic extract of *C. reticulata* leaves. The investigation resulted in the discovery of a new acridone alkaloid, named reticarcidone A (**205**), being the first pyrano of the *Citrus* genus. In addition, nine already known acridone alkaloids and fifteen flavones were isolated from this same extract. Some of these compounds showed moderate cytotoxicity against five human cancer cell lines (breast, liver cancer, promyelocytic leukemia, lung cancer, and colon cancer). These results expand the knowledge about the chemical diversity of *C. reticulata* and indicate a promising potential for anticancer pharmacological applications.

Research suggests that citrus fruit residues, such as peels and leaves, contain bioactive compounds with the ability to create protective barriers on metal surfaces, contributing to a decrease in the corrosion rate of metallic compounds. Najem et al. [55] showed an inhibition rate of 90.13% for carbon steel in oil formation water, suggesting the physical adsorption of active components on the steel surface. Thus, it is reasonable to assume the promising use of these residues as efficient and environmentally sustainable corrosion inhibitors.

The extract from *C. reticulata* stem bark demonstrated significant cytotoxic activity against lung cancer (A549), breast (MCF7), and prostate (PC3) cells, with low IC_50_ values, especially in MCF7 cells. A fraction rich in compounds such as scoparone (**86**), xanthyletin (**87**), lupeol (**79**), and plant sterols showed strong cytotoxic action and selectivity, minimally affecting normal cells. The results suggest a promising anticancer potential, possibly associated with synergistic effects between the isolated compounds, as exposed by Tahsin et al. [24].

Srimathi et al. [59] demonstrated that naringenin (**104**), a flavonoid isolated from *C. reticulata* peels, shows significant potential in neutralizing the toxic effects of *N. naja* cobra venom. In in vitro tests, naringenin (**104**) inhibited 51% of PLA_2_ enzyme activity and reduced venom‐induced hemolytic activity by 59.8%. In in vivo experiments, performed in murine models, significant reductions in inflammatory processes, such as edema, were observed, in addition to protective effects on tissues, evidenced by histopathological analyses.

Castro et al. [18] demonstrated that the EO extracted from the fruit peels significantly inhibits the proliferation of non‐small cell lung cancer cells. The oil reduced cell growth in a dose‐dependent manner. The observed mechanisms included cell cycle arrest in the G_0_/G_1_ phase, reduction of membrane‐bound Ras protein, and induction of apoptosis. Furthermore, no signs of hepatic toxicity were identified.

The insecticidal activity of EOs against *Thrips flavus* was associated with their chemical composition. The EO from *C. reticulata* leaves, rich in monoterpenes, and from the peels, with a high content of *D*‐limonene (**3**), showed 100% pest control. Linalool (**19**) showed good toxicity and attractiveness, suggesting behavioral action. These results indicate that compounds such as *D*‐limonene (**3**) and linalool (**19**) are responsible for the efficacy of these oils, reinforcing their potential in natural pest control, narrates Pei et al. [60].

## 4 Future Perspectives

- Formulation of nanoparticles containing limonene or flavonoids from the peels for transdermal or oral application with controlled release;
- Potential application: prevention of chronic diseases through the modulation of signaling pathways such as NF‐κB, PI3K/Akt, and MAPK;
- Nutraceutical formulations with standardized extracts aiming at neuronal protection and clonal anti‐inflammatory activity;
- Use in medical surface coatings, mouthwashes, and natural preservatives through biofilm control;
- Use of agricultural waste (peels and leaves) in the formulation of products for organic agriculture (biopesticides);
- Incorporation into topical emulsions and anti‐acne products with a natural and sustainable appeal;
- Genetic modification of microorganisms for potentiation of *C. reticulata* Blanco bioactive compounds.

## 5 Registration of Patents for *Citrus reticulata* Blanco

The World Intellectual Property Organization (WIPO) is included in the global sharing of registered patents. WIPO data concerning the *C. reticulata* Blanco species comprises its use as food, insecticidal activity, anti‐inflammatory, and antioxidant activities (Table 2).

**TABLE 2:** Registration of patents *Citrus reticulata* Blanco.

Country applicant	Plant part isolated	Title/Application	International classification
China	Fruits	Preparations for teas, liqueurs, and wines, and nutritious flours, for example, with additives (flavoring) food preservation (composition, freezing; subsequent thawing; chilling) Phytotherapeutic baths	A23F 3/14 A23B 9/00 A23B 7/04 A23L 2/02 A23L 2/06 A23L 2/52 A23L 7/10 A23L 27/60 A23K 10/37 A23L 2/02 A23L 2/52 A23F 3/34 A61K 36/80 A61P 11/14 A61P 11/10 A23F 3/34 A23L 5/20 A23L 3/3472 A23F 3/14 A23F 3/06 A61K 36/8998 A61P 1/14 A61P 7/06 A61K 36/82 A61K 36/79 A61K 36/62 A61K 36/73 A61K 36/725 A61P 25/00 A61L 9/013 A61L 101/56 C12G 3/055 C12G 3/05 A61K 36/8967 A61P 37/04 A61P 1/14 A61P 1/16 C07H 15/18 A23L 1/068 C12N 9/99 A61K 31/715 A61P 43/00 A23F 3/34 A61K 36/8969 A61K 36/481 A61K 36/815 A61K 36/258 A61P 1/14 A23L 2/02 A23L 2/52 A23L 33/105 A61K 36/888 A61K 36/88 A61P 39/06 A61P 17/16 A61P 17/00 A61P 29/00 C12G 1/00 C12G 3/055 B67B 7/04 B65G 65/40 A23K 50/10 A23K 10/30 A23K 40/00 A23F 3/14 A23P 20/25 A23L 2/02 A23L 2/52 A23L 33/105 A61K 36/884 A61K 36/752 A61K 36/734 A61K 36/708 A61K 36/704 A61K 36/62 A23L 33/00 A61K 36/8994
China	Seedlings	Mold prevention	A01G 13/00 A01G 2/30 A01N 59/20 A01N 59/06 A01P 3/00 A01D 46/24 C05G 3/00 A01G 1/00 A01G 9/10 A01G 23/04 A01G 9/029 A01G 22/30 A01G 7/06 A01G 27/06
China	Complete plant	Vegetative propagation (reproduction of plants by tissue culture techniques)	A01G 2/00
China	Biomass	Compositions of solid adsorbents or compositions of filtration aids; sorbents for chromatography; processes for preparing, regenerating, or reactivating them	B01J 20/20 B01J 20/30 C02F 1/28 C02F 101/20
China	Fruits	Manures, seeders, and fertilizers	A01C 21/00 C05F 15/00 C05G 1/00 C05F 17/20 A23K 1/14
China	Plant extract	Modifying the nutritive qualities of foodstuffs; dietetic products; preparation or treatment thereof, uric acid reduction	A61K 36/752 A23L 33/105 A61P 19/06 A61P 13/04 A61P 13/12 A61P 19/02 A23L 33/00 A23L 33/105 A23L 33/135 A23L 33/10 A23L 33/19 A23P 10/30
China	Peels	Medicines for dermatological disorders	A61K 36/752 A61P 17/02 A61K 7/06 A61K 31/4433 A61K 35/56 A61K 35/78 A61P 17/14 A61K 7/00 A61K 7/48 A61K 35/78 A61K 7/06 A61K 7/50 A61K 8/9789 A61K 8/49 A61Q 19/00 A61Q 19/02 A61K 36/752 A61P 17/02 A61P 17/00 A61P 37/04 A61K 8/92 A61Q 19/00 A61K 36/9068 A61K 9/48 A61P 17/00 A61P 37/08 A61K 35/64 A61K 35/62 A61K 36/8969 A61P 3/04 A61P 3/06 A61K 35/64
República da Coreia	Fruits	Anticancer, anti‐inflammatory, hepatoprotective, antiulcerative, and antimicrobial therapeutic agent	A61K 36/752 A61P 35/00 A61P 29/00 A61K 36/752 A61P 1/16 A61P 1/00 A61P 3/00 A61K 36/8994 A61P 11/00 A61P 11/14 A61K 36/8905 A61K 08/09 A61H 39/06 A61P 15/08 A61K 35/32 A61K 35/50 A61K 36/9068 A61P 1/04 A61K 33/26 A61K 36/9064 A61P 1/16 A61K 36/752 A61P 1/02 A61P 31/10 A61P 29/00 A61P 39/02 A61P 1/14 A61K 36/808 A61P 11/00 A61K 35/64 A61K 36/904 A61K 9/20 A61P 11/06 A61P 11/14 A61P 11/00 A61K 33/06 A61K 36/9064 A61P 1/14 A61K 35/57 A61K 36/9066 A61P 9/10 A61K 35/32 A61K 35/36 A61K 35/56 A61K 35/62 A61K 36/8905 A61P 1/16 A61K 36/8964 A61P 35/00 A61K 35/64 A61K 36/9068 A61P 1/00 A61K 36/815 A61K 9/20 A61P 27/02 A61P 1/16 A61P 7/06 A61P 1/14 A61K 36/78 A61K 36/8994 A61K 36/899 A61K 36/8966 A61K 16/09 A61K 06/09 A61K 36/904 A61P 11/00 A61P 11/06 A61P 11/10 A61P 11/14 A61K 36/9066 A61P 15/00 A61K 35/618 A61K 35/24 A61K 36/752 A61K 36/746 A61K 36/64 A61K 36/54 A61K 36/076 A61K 35/56 A61K 36/904 A61P 31/04 A61K 35/36 A61K 35/618 A61K 36/8968 A61K 36/8888 A61K 36/752 A61K 36/736 A61K 36/605 A61K 36/539 A61K 36/9068 A61K 14/09 A61K 16/09 A61K 9/20
			A61K 9/48 A61P 11/02 A61K 36/9066 A61K 9/20 A61P 19/08 A61K 35/50 A61K 35/64 A61K 36/752 A61P 17/02 A61K 36/904 A61K 9/20 A61K 06/09 A61P 11/00 A61P 11/06 A61K 36/8888 A61P 1/02 A61K 36/8945 A61P 11/06 A61K 35/644 A61K 35/64
China	Fruits	Nonalcoholic beverages; dry compositions or their concentrates	A23L 2/02 A23L 2/84 A23L 33/105 C12N 1/14 C12P 19/14 C12P 19/60
China	Pericarp	Probiotic activity	A23L 33/105 A23L 33/10 A23L 33/135 A23P 28/10 A61K 8/9789 A61K 8/99 A61K 36/752 A61Q 11/00 A61P 1/02 A61P 31/02 A23F 3/14 A61K 36/8969 A61P 1/14 A61P 1/10 A61K 35/741 A61K 35/745
China	Pericarp	Medicinal preparations characterized by a special physical form (cleaning, sterilization, and disinfection)	A61K 36/752 A61K 9/12 A61K 08/09 A61P 11/14 A61P 11/10 A61P 1/02
China	Peel	Drugs for sensory disorders (pulmonary embolism)	A61K 36/88 A61P 27/02 A61P 7/02 A61P 9/10
China	Peel	Processes, in general, for the preparation of catalysts; processes, in general, for the activation of catalysts (biodiesel)	B01J 37/00 C07C 67/00 C10L 1/00 D21C 3/04 D21C 3/02 D21B 1/06
Japão	Lyophilized	Biocides, repellents, or pest attractants or growth regulators for plants (Insecticide)	A01N 65/00 A01P 3/00 A01P 7/04
		Compounds containing saccharide radicals	

The *C. reticulata* species has been widely explored in various technological fronts, as evidenced by the patent survey that demonstrates its multifunctional potential. The identified applications range from the food industry to pharmaceutical, agricultural, and industrial sectors, highlighting the versatility of the compounds present in this species.

In the food sector, preparations for teas, liqueurs, wines, and flours enriched with flavoring additives stand out. Applications are also reported in food preservation processes, including compositions for freezing, defrosting, and chilling, as well as formulations for nonalcoholic beverages and dietary products with a focus on modifying nutritional properties, such as uric acid reduction.

In the health field, medicinal applications are numerous, including formulations with anticancer, anti‐inflammatory, hepatoprotective, antiulcerative, antimicrobial, and probiotic therapeutic activity. Patents also describe the use of the plant in the treatment of dermatological and sensory disorders, such as in the case of pulmonary embolism, as well as in specific medicinal preparations for cleaning, sterilization, and disinfection.

In phytotherapy, the use of *C. reticulata* in therapeutic baths is noteworthy, whereas in the field of plant biotechnology, its application in vegetative propagation techniques by tissue culture is observed. From an agricultural and environmental perspective, the species is applied as fertilizer and soil amendment, in addition to comprising formulations for biocides, repellents, pest attractants, and plant growth regulators, with emphasis on its insecticidal action.

In the field of chemistry and materials engineering, uses emerge in compositions of solid adsorbents and filtration aids, including in chromatography, as well as in processes for the preparation and activation of catalysts, with potential application in biodiesel production. The presence of compounds containing saccharide radicals also opens up space for investigations in fine chemistry and biotechnology. This set of applications reinforces the strategic value of *C. reticulata* as a source of technological innovation, with concrete possibilities for the development of new products and processes in the most diverse areas.

## 6 Conclusion

The different parts of the plant *C. reticulata* Blanco exhibit a wide range of biological properties, with particular emphasis on the fruit peels, where most secondary metabolites are concentrated. Among these compounds, flavonoids constitute the main nonvolatile components, whereas terpenes represent the predominant volatile compounds, both widely recognized for their multiple pharmacological activities.

The species *C. reticulata* stands out as a promising natural resource for the development of new therapeutic alternatives, especially in the face of the growing challenge of antimicrobial resistance to conventional treatments. In addition, it shows potential in combating oxidative stress, allergic reactions, and skin disorders associated with the action of free radicals. Its anti‐inflammatory properties are related to the modulation of cellular signaling and cell cycle regulators, further reinforcing its biotechnological relevance.

Future research should focus on elucidating the molecular mechanisms underlying the species’ antibiotic and antioxidant potential, as well as its possible role in modulation processes. Therefore, it is essential to explore its applications in improving the nutritional quality of foods, developing dietary products, controlling pests, and formulating natural fertilizers, always prioritizing safe and non‐toxic medicinal use.

## Author Contributions

**José Walber Gonçalves Castro**: research, writing, formatting, chemical structure, and editing. **Geane Gabriele de Oliveira Souza**: research, writing, formatting, and editing. **José Galberto Martins da Costa**: conception of the idea and revision. **Fabíola Fernandes Galvão Rodrigues**: conception of the idea and revision.

## Conflicts of Interest

The authors declare no conflicts of interest.

## Data Availability

The data that support the findings of this study are available from the corresponding author upon reasonable request.
